# Recurrent herpes simplex virus-1 infection induces hallmarks of neurodegeneration and cognitive deficits in mice

**DOI:** 10.1371/journal.ppat.1007617

**Published:** 2019-03-14

**Authors:** Giovanna De Chiara, Roberto Piacentini, Marco Fabiani, Alessia Mastrodonato, Maria Elena Marcocci, Dolores Limongi, Giorgia Napoletani, Virginia Protto, Paolo Coluccio, Ignacio Celestino, Domenica Donatella Li Puma, Claudio Grassi, Anna Teresa Palamara

**Affiliations:** 1 Institute of Translational Pharmacology, National Research Council, Rome, Italy; 2 Institute of Human Physiology, Università Cattolica del Sacro Cuore, Rome, Italy; 3 Fondazione Policlinico Universitario A. Gemelli IRCCS, Rome, Italy; 4 Department of Public Health and Infectious Diseases, Sapienza University of Rome, Laboratory affiliated to Istituto Pasteur Italia–Fondazione Cenci Bolognetti, Rome, Italy; 5 San Raffaele Pisana, IRCCS, Telematic University, Rome, Italy; University of Wisconsin-Madison, UNITED STATES

## Abstract

Herpes simplex virus type 1 (HSV-1) is a DNA neurotropic virus, usually establishing latent infections in the trigeminal ganglia followed by periodic reactivations. Although numerous findings suggested potential links between HSV-1 and Alzheimer’s disease (AD), a causal relation has not been demonstrated yet. Hence, we set up a model of recurrent HSV-1 infection in mice undergoing repeated cycles of viral reactivation. By virological and molecular analyses we found: i) HSV-1 spreading and replication in different brain regions after thermal stress-induced virus reactivations; ii) accumulation of AD hallmarks including amyloid-β protein, tau hyperphosphorylation, and neuroinflammation markers (astrogliosis, IL-1β and IL-6). Remarkably, the progressive accumulation of AD molecular biomarkers in neocortex and hippocampus of HSV-1 infected mice, triggered by repeated virus reactivations, correlated with increasing cognitive deficits becoming irreversible after seven cycles of reactivation. Collectively, our findings provide evidence that mild and recurrent HSV-1 infections in the central nervous system produce an AD-like phenotype and suggest that they are a risk factor for AD.

## Author summary

This paper provides novel evidence that herpes simplex virus type-1 infection in mice, undergoing repeated virus reactivations throughout life, as occurs in those humans suffering of recurrent infection, triggers progressive accumulation of molecular markers of neurodegeneration and cognitive decline that are reminiscent of Alzheimer’s disease phenotype.

## Introduction

Herpes simplex virus type 1 (HSV-1) is a neurotropic virus that establishes a latent infection in sensory, typically the trigeminal, ganglia and periodically reactivates giving rise to the well-known cold sores and blisters on orolabial mucosa [1]. Following reactivation, HSV-1 may also reach the central nervous system (CNS) [2–5] resulting in either a severe, but rare, form of herpetic encephalitis (HSE) or establishing latency [6, 7]. Increasing evidence suggests a possible link between HSV-1 infection and Alzheimer’s disease (AD), the most common neurodegenerative disease associated with dementia in the elderly [8–10]. Previous studies demonstrated the presence of HSV-1 genome in the brain of AD patients, particularly those carrying the ε4 allele of apolipoprotein E that is a risk factor for AD [11–13]. More recently, population-based studies correlated the level and avidity index of anti-HSV-1 IgG and IgM (markers of HSV-1 infection and reactivation, respectively) to the risk of developing AD [14–18]. Many *in vitro* studies including ours supported the hypothesis that HSV-1 is involved in AD pathogenesis. Specifically, we demonstrated that HSV-1 infection in cultured neurons induces the amyloidogenic processing of amyloid precursor protein (APP) and intra- and extra-neuronal accumulation of amyloid-β protein (Aβ) and other neurotoxic APP fragments [19, 20]. We also demonstrated that HSV-1 activates intracellular processes leading to neurodegeneration through different mechanisms, most of them driven by APP fragments [21–23]. Others groups pointed out the effects of HSV-1 infection on the microtubule-associated protein tau, showing that the virus triggers its hyperphosphorylation and aggregation, likely leading to its deposition in neurofibrillary tangles (NFTs), another hallmark of AD [24, 25]. Altogether these data strongly support the hypothesis that accumulation of AD biomarkers triggered by recurrent infection, if not properly cleared, may drive neurodegeneration. This view was supported by the results of *in vivo* studies showing inflammatory and neurodegenerative markers in a mouse model of HSE induced by intranasal HSV-1 inoculation [26]. However, a clear cause-effect relationship among multiple HSV-1 reactivations, active viral replication in the brain, accumulation of AD molecular markers and cognitive deficits has yet to be demonstrated. This issue is very relevant for personalized medicine approaches when considering the role of microbial agents in neurodegenerative diseases. Hence, we established a mouse model of HSV-1 infection and reactivation to verify whether multiple viral reactivations throughout life result in an AD-like phenotype. Following virus reactivations, we found that: i) HSV-1 actively replicated in the brain; ii) AD biomarkers progressively accumulated in neocortex and hippocampus of infected mice; iii) AD biomarker accumulation was associated with cognitive impairment. These biochemical and functional alterations increased with the number of virus reactivations.

## Results

### Experimental model of HSV-1 infection and reactivation in mice

**Fig 1:**
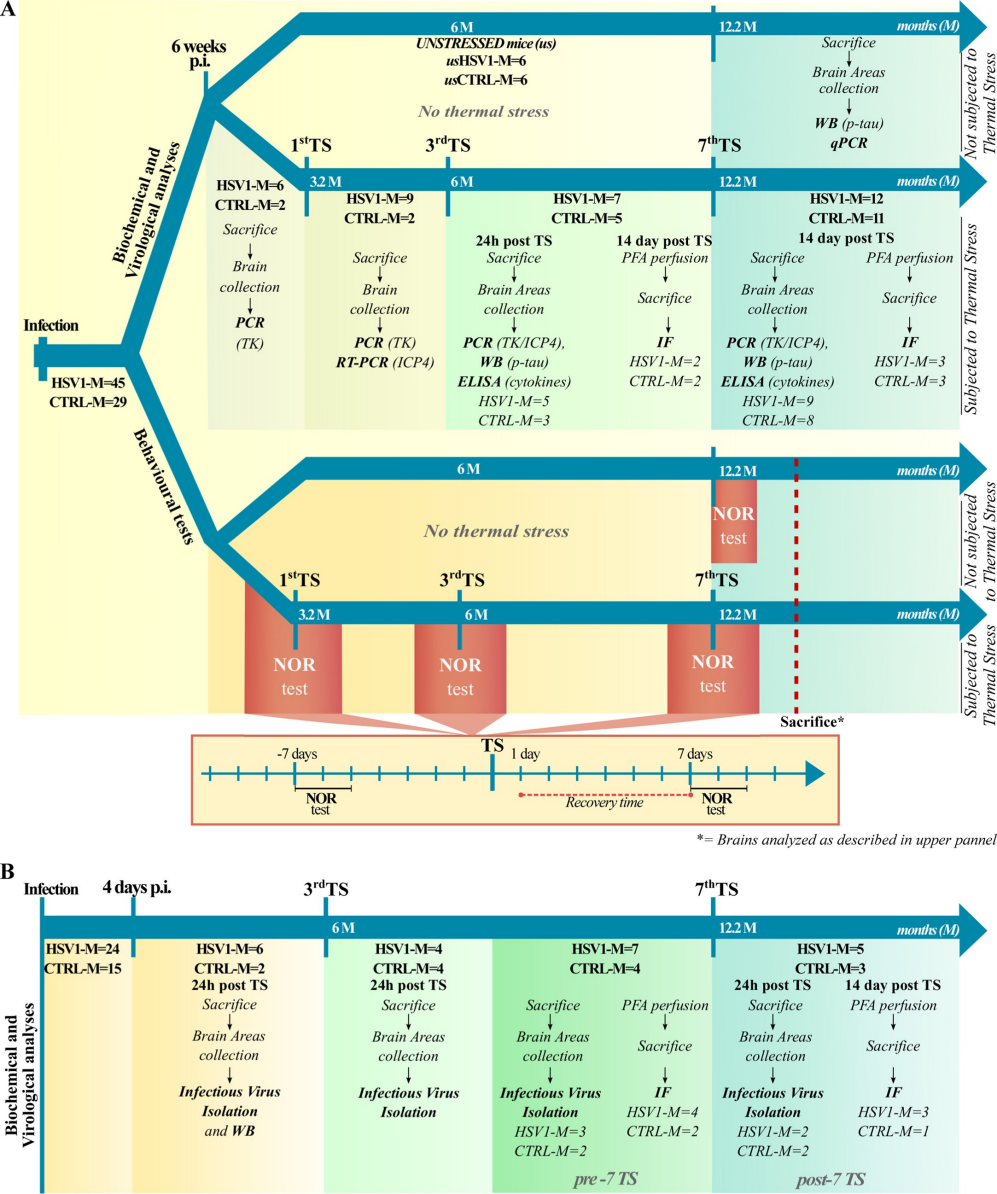
Schematic representation of the experimental design. (A) Timeline of biochemical and molecular analyses: 6–8 week-old BALB/c mice were infected with HSV-1 (n = 45, HSV1-M) or mock solution (n = 29, CTRL-M); 6 CTRL-M and 6 HSV1-M were separated 6 weeks post infection (p.i.) as UNSTRESSED group (named hereafter usHSV1-M and usCTRL-M, see upper arrow), the other mice undergone several cycles of thermal stress (TS) as described in Methods (bottom arrow); one day before the 1^st^ TS (6 weeks p.i.), 6 HSV1-M and 2 CTRL-M were sacrificed and their brain samples were collected for PCR analysis. Similarly, other mice were sacrificed, and their brain samples collected 24 h post the 1^st^ TS (n = 9 HSV1-M, n = 2 CTRL-M), the 3^rd^ TS (n = 5 HSV1-M, n = 3 CTRL-M) and following the 7^th^ TS (n = 9 HSV1-M, n = 8 CTRL-M). Brain areas from 3 TK^+^ HSV1-M sacrificed 24 h post the 3^rd^ TS, and from 4 TK^+^ HSV1-M sacrificed 14 days post the 7^th^ TS were used for WB and ELISA assay. Matched CTRL-M were similarly analyzed. Fourteen days post the 3^rd^ TS (n = 2) and the 7^th^ TS (n = 3) the indicated HSV1-M were perfused with PFA and their brain slices analyzed by IF. Matched CTRL-M were sacrificed for the same analyses. Timeline of behavioral tests: UNSTRESSED mice were tested in NOR 11 months p.i., and then sacrificed for biochemical analyses (upper arrow); 7 days before and after the indicated TS the same subset of 9 HSV1-M and 9 CTRL-M were subjected to NOR test (lasting 3 days), and then sacrificed for biochemical and molecular analyses as indicated above (bottom arrow). (B) 6–8 week-old BALB/c mice were infected with HSV-1 (n = 24, HSV1-M) or mock solution (n = 15, CTRL-M) under the same experimental conditions of A. Four days p.i., 6 HSV1-M and 2 CTRL-M were sacrificed and their lips, TGs and bran tissues collected and analyzed by virological techniques for the presence of the virus; TGs were also assessed by WB for ICP4 presence. The other mice undergone several cycles of TS as for mice described in A. Infectious virus isolation was performed from TGs and brain areas from 4 HSV1-M and 4 CTRL-M sacrificed 24 h post the 3^rd^ TS and titered by SPA post incubation on VERO cells, as described in methods. Infectious virus isolation was performed also from TGs and brain areas from 3 HSV1-M and 2 CTRL-M sacrificed before the 7^th^ TS and 2 HSV1-M and 2 CTRL-M sacrificed 24h post the 7^th^ TS. Their titers were directly assessed by ICW assay. Fourteen days after the 7^th^ TS, 3 HSV1-M were perfused with PFA (post-7TS). In parallel, 4 HSV1-M that did not undergo the 7^th^ TS were perfused with PFA (pre-7TS). Brain slices from pre- and post-7TS mice were then analyzed by IF for gB expression and phospho-tau (p-tau) levels. Matched CTRL-M were parallel analyzed in IF.

To establish the mouse model of recurrent HSV-1 infection, 74 anesthetized 6-8-week-old BALB/c mice were inoculated with either HSV-1 (HSV1-M; n = 45) or mock solution (CTRL-M; n = 29) via lip scarification. After 6 weeks, 6 HSV1-M and 6 CTRL-M were kept unstressed until 11 months post infection (p.i.) (named hereafter as UNSTRESSED group, i.e., usHSV1-M and usCTRL-M, respectively), whereas the others were subjected to hyperthermia to induce the reactivation of latent virus, essentially based on previously reported protocols [27] (see timeline of experiments in Fig 1A). Specifically, mice underwent 15 min thermal stress (TS) in a 43°C water bath and then they were placed under a warm lamp for 30 min to prevent hypothermia, as described in Methods. TS was repeated at 6–8 week intervals, up to 7 times, in order to obtain multiple viral reactivations. This protocol of virus infection and reactivation was set up on the basis of preliminary experiments in which we tested different HSV-1 doses (10^5^−10^8^ plaque forming unit [PFU] range) and inoculation procedures (lip scarification and intraperitoneal injection) to determine the most efficient conditions that promoted virus spreading to the CNS avoiding the occurrence of virus-induced encephalitis with seizures or paralysis. Under the chosen experimental conditions, HSV1-M and CTRL-M did not show any differences in body weight over life (S1A Fig).

**Fig 2:**
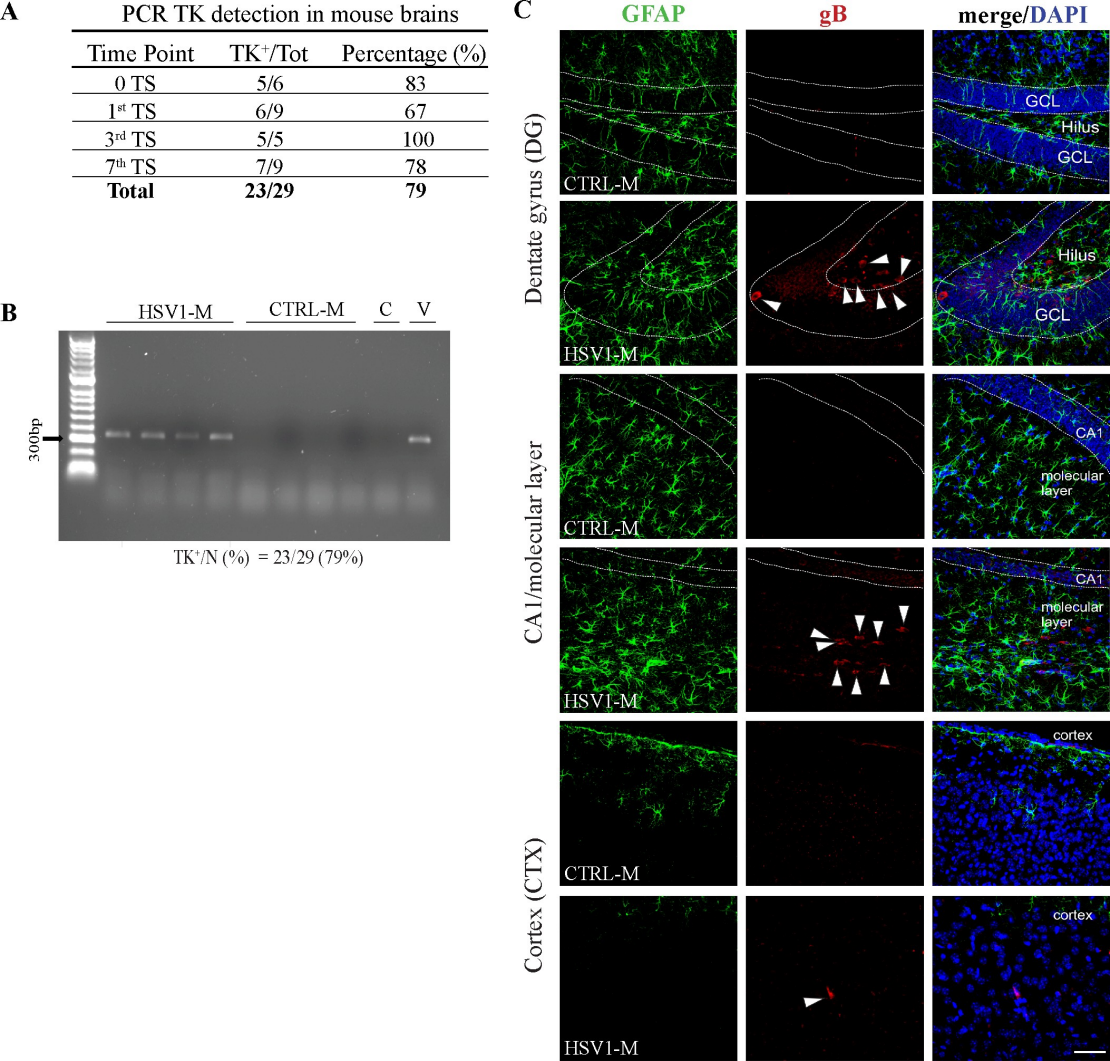
Presence of HSV-1 in the brain. (A) Table summarizing the percentage of TK^+^ brains at the indicated time points as found with PCR performed on subsets of mice sacrificed 6 weeks p.i. (0 TS), and following the 1^st^, 3^rd^, and 7^th^ TS. These analyses were performed on DNA isolated from whole brain or from specific cerebral regions: those brains showing TK gene in at least one region were considered positive. (B) Representative image of PCR amplification of TK gene in 4 out of 29 HSV1-M and the corresponding CTRL-M. C = negative control of PCR reaction; V = HSV-1 TK amplification, as positive control. The total number of the studied brains and the percentages of TK^+^ brains are shown under the gel. (C) Confocal immunofluorescence analysis of gB and Glial Fibrillary Acidic Protein (GFAP) expression in brain slices from HSV1-M (n = 3) and CTRL-M (n = 2) sacrificed following the 7^th^ TS. Two randomly selected coronal sections were analyzed for each brain. Representative images of different areas within hippocampus (DG = dentate gyrus, CA1/molecular layer), and somatosensory neocortex (CTX) are shown. Nuclear bodies were stained by DAPI. Dotted lines delimitate pyramidal neuron layer in CA1 and granule cell layer (GCL) from hilus in DG. Arrowheads indicate gB^+^ cells. Scale bar: 50 μm.

To check virus spreading to the brain, 6 HSV1-M were sacrificed 6 weeks p.i., and viral TK gene was amplified by PCR on DNA extracted from their brain tissues. Five of the 6 brains were TK^+^, indicating that the first infection was sufficient to determine virus spreading to the brain in a high percentage (83%) of animals (Fig 2A). Similar results were obtained from brain tissues of HSV1-M (n = 23) sacrificed at different time points after TS (representative gel in Fig 2B). Viral TK gene was found in 79% of HSV1-M, thus confirming the presence of HSV-1 in the brain of most studied animals. The specific percentages of TK^+^ brains from mice sacrificed 6 weeks p.i. and then after the first, the third and the seventh TSs are shown in the table in Fig 2A. It is noteworthy to mention that brains found negative for HSV-1 may be related to different factors, including individual variability in virus spreading to the brain, failure in HSV-1 primary infection, technical problems in DNA extraction or amplification. However, given our interest to study the effects of multiple HSV-1 replications in the brain, we limited our further analyses to mice exhibiting HSV-1^+^ brains.

Then, HSV1-M and CTRL-M brains were analyzed for mRNA of ICP4 (the main transcriptional HSV-1 regulatory protein), that was found in productive and abortive virus reactivation [28, 29], and the expression of late viral gB protein. ICP4 mRNA was found in 61% of the analyzed TK^+^ brains (n = 18, representative image in S1B Fig). In addition, gB^+^ cells were detected in the brains of all the analyzed HSV1-M sacrificed after the third (n = 2, S1C Fig) and the seventh TSs (n = 3, Fig 2C).

**Fig 3:**
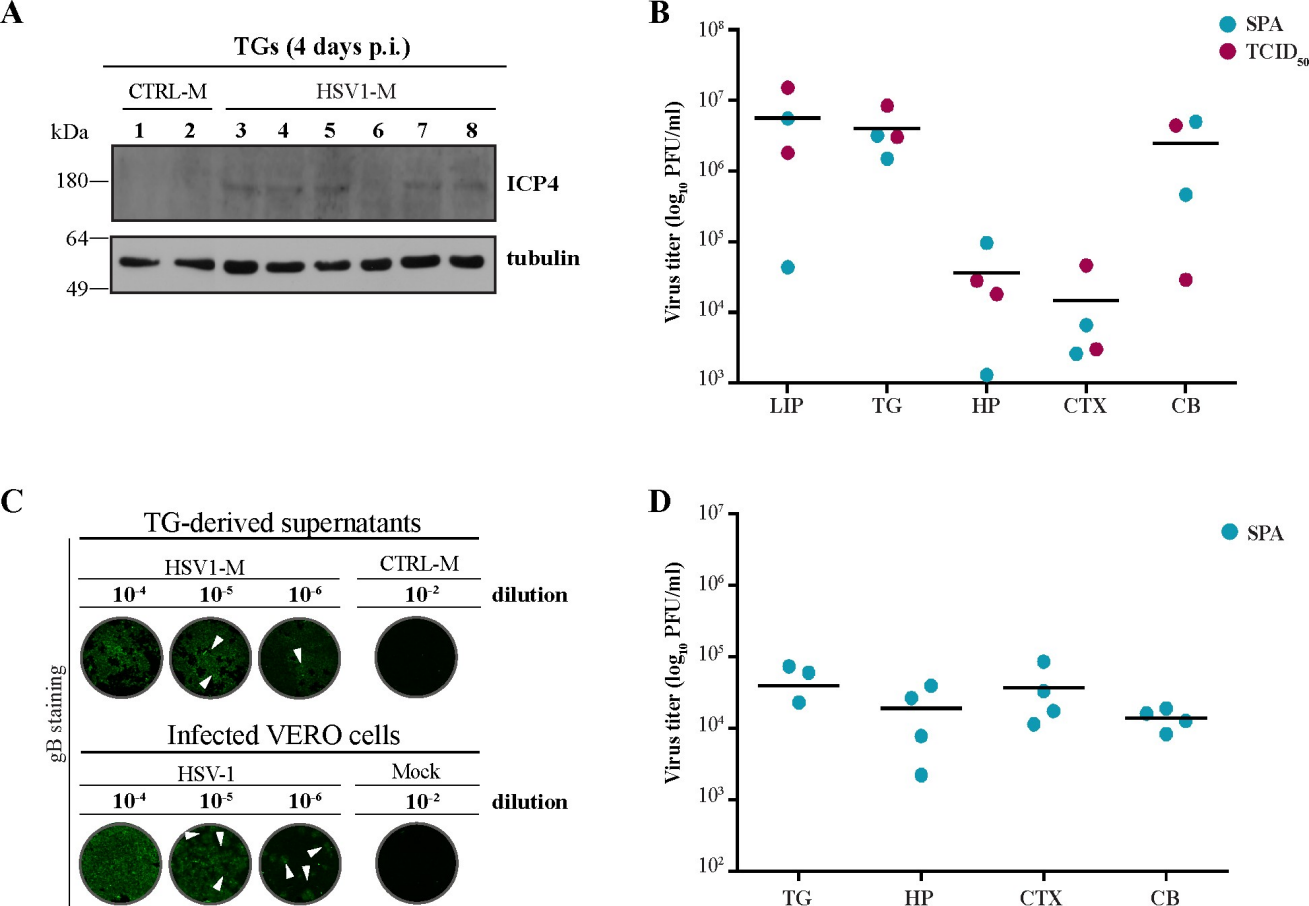
Efficacy of virus inoculation and reactivation in mice. (A-C) Efficacy of virus inoculation. (A) Western blot showing the presence of ICP4 in trigeminal ganglia (TGs) from HSV1-M (n = 6) and CTRL-M (n = 2) sacrificed 4 days p.i.; tubulin expression level was used as sample loading control. (B) Lip, TG, hippocampus (HP), cortex (CTX) and cerebellum (CB) from HSV1-M were homogenized as described in Methods, and incubated on VERO cells for 3 h, then replaced with complete RPMI. Four days p.i., cell supernatants were harvested and then assayed on VERO cells by standard plaque assay (SPA, n = 2 for each tissue) and Reed and Muench method (TCDI50, n = 2 for each tissue) to evaluate virus titer. Scatter dot plot shows the individual and mean values of virus titer (log_10_ PFU/ml) assayed by SPA (blue dots) and TCID_50_ (purple dots). (C) In Cell Western (ICW) assay, upper image: after 3h of incubation with the indicated serial dilutions of TG-derived supernatants (TG-derived supernatants, n = 2, obtained as described in B), VERO cells were left for 72 h with RPMI 2% FBS and then fixed and stained with anti-gB antibody (green, upper wells); lower image: the same staining was performed on VERO cells infected for 24 h with stock HSV-1 or mock solution at the indicated dilutions (Infected VERO cells). Arrowheads indicate representative gB^+^ foci of infection. (D) TG and the indicated brain tissues were harvested from 4 HSV1-M sacrificed 24h after the 3^rd^ TS, homogenized as described in Methods, incubated on VERO cells for 3 h, then replaced with complete RPMI. Four days p.i., cell supernatants were harvested and then assayed on VERO cells by SPA. Scatter dot plot shows the individual and mean values of virus titer (log_10_ PFU/ml) assayed by SPA (blue dots).

To further characterize this *in vivo* model, a new cohort of 39 animals was HSV-1- (n = 24) and mock- (n = 15) infected and then subjected to TS under the same experimental conditions used for mice of the first cohort (see timeline of experiments in Fig 1B). In these mice we also checked the occurrence of virus infection and its spreading to trigeminal ganglion (TG), as well as the efficacy of TS in inducing virus replication. Thus, we first assessed the expression of viral ICP4 in the TGs obtained from a subset of mice sacrificed 4 days p.i.. Western blot (WB) analysis showed a slight 180 kDa band recognized by anti-ICP4 antibody only in the TGs from HSV1-M (Fig 3A), thus indicating that infection had been established. To confirm this result, infectious virus was then isolated from homogenized TGs and, after a first round of infection on VERO cells, it was titered with different virological methods (Fig 3B and 3C). Specifically, virus titer in the cell supernatants was assessed by standard plaque assay (SPA, 2 TGs) [30], Reed and Muench method (TCID_50_, 2 TGs) [31], and In Cell Western assay (ICW, 2 TGs) [32], that check the expression of gB protein in infected cells. Similarly, infectious virus was titered in supernatants derived from VERO cells previously infected with homogenized lip, hippocampus (HP), neocortex (CTX), and cerebellum (CB) from the same mice (Fig 3B). Results indicated that 100% of mice were infected following HSV-1 lip-inoculation, and that virus could reach CNS following labial inoculation.

Next, to check the effect of TSs on virus reactivation, we first assessed the presence of infectious virus in homogenized mouse tissues collected from a subset of mice (n = 4 for each experimental group) sacrificed 24 hours (h) after the third TS. These homogenates were first incubated on VERO cells for 3 h and the virus released in cell supernatants after 4 days was quantified through SPA (Fig 3D). Then, a subset of HSV1-M and CTRL-M were sacrificed before (pre-TS) and after (post-TS) the seventh TS to further check whether virus replication in the brain was TS-dependent. To this aim, some of these mice were used for immunofluorescence (IF) analysis of gB expression in the brain, the others for detection and titration of infectious virus in their tissues. Results in S2A Fig showed that gB was detectable in brain slices from HSV1-M that were sacrificed post-TS (n = 3), but not in those analyzed pre-TS (n = 4), indicating that actually TS was able to induce virus replication in the brain. Accordingly, direct ICW assay (performed with serial dilutions of tissue homogenates as infectious solution) revealed a higher titer of infectious virus in TG and brain from HSV1-M sacrificed post-TS (n = 2) with respect to those from mice sacrificed before TS (n = 3, S2B Fig). These results confirmed the occurrence of active virus replication post-TS.

Collectively, our data indicate that in our experimental model HSV-1 reaches the brain, where it actively replicates after TS-induced reactivations.

### Neuropathological changes induced by recurrent HSV-1 infection in mice

Next, we characterized this mouse model at molecular level. In particular, we checked the appearance of Aβ, that we previously detected in cultured neurons subjected to acute HSV-1 infection [19–23], the pattern of tau phosphorylation, and signs of neuroinflammation (e.g., proinflammatory cytokine levels and astrocytosis). For biochemical analyses we used HSV-1^+^ brains. Specifically, neocortices and hippocampi from HSV1-M and CTRL-M were analyzed after the third TS (n = 5 for each experimental group), and the seventh TS (n = 7 for each experimental group) by WB, IF, and immunoperoxidase staining (IPS). WB analyses were also performed on usHSV1-M and usCTRL-M (the UNSTRESSED group of mice), whereas IF analyses were performed also on the subset of animals sacrificed before and after the seventh TS. The experimental design and timeline of these experiments are shown in Fig 1.

**Fig 4:**
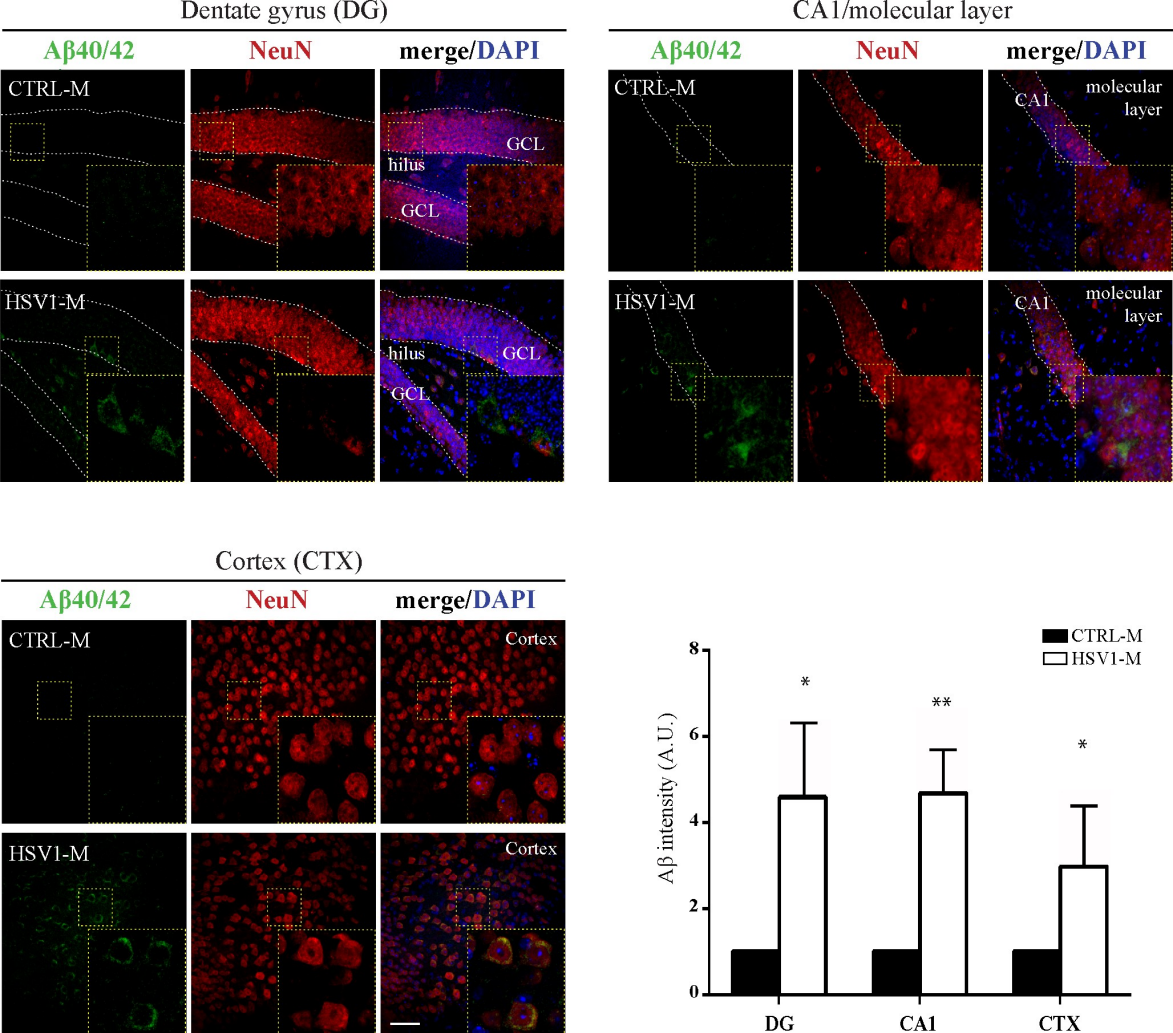
Multiple HSV-1 reactivations induce increased Aβ accumulation. Confocal immunofluorescence analysis of coronal brain slices from HSV1-M and CTRL-M undergone 7 TSs (n = 3 mice for each experimental group). Two/three coronal sections were analyzed for each brain. Aβ40/42 were recognized by immunoreactivity for a specific antibody (see S7B Fig). Neurons were identified by their immunoreactivity for anti-NeuN antibody. Cell nuclei were stained with DAPI. Panels show representative images from dentate gyrus (DG), hippocampal CA1 (including the molecular layer) and somatosensory neocortex (CTX). Insets show higher magnification (4×) of boxes outlined in each panel. Dotted lines delimitate pyramidal neuron layer in CA1 and granule cell layer (GCL) from hilus in DG. Scale bar: 50 μm. Bar graphs showing mean Aβ fluorescence intensity quantified in the studied brain areas and expressed as fold change with respect to CTRL-M. Data are represented as: mean ± SEM, * p<0.05, ** p<0.01.

**Fig 5:**
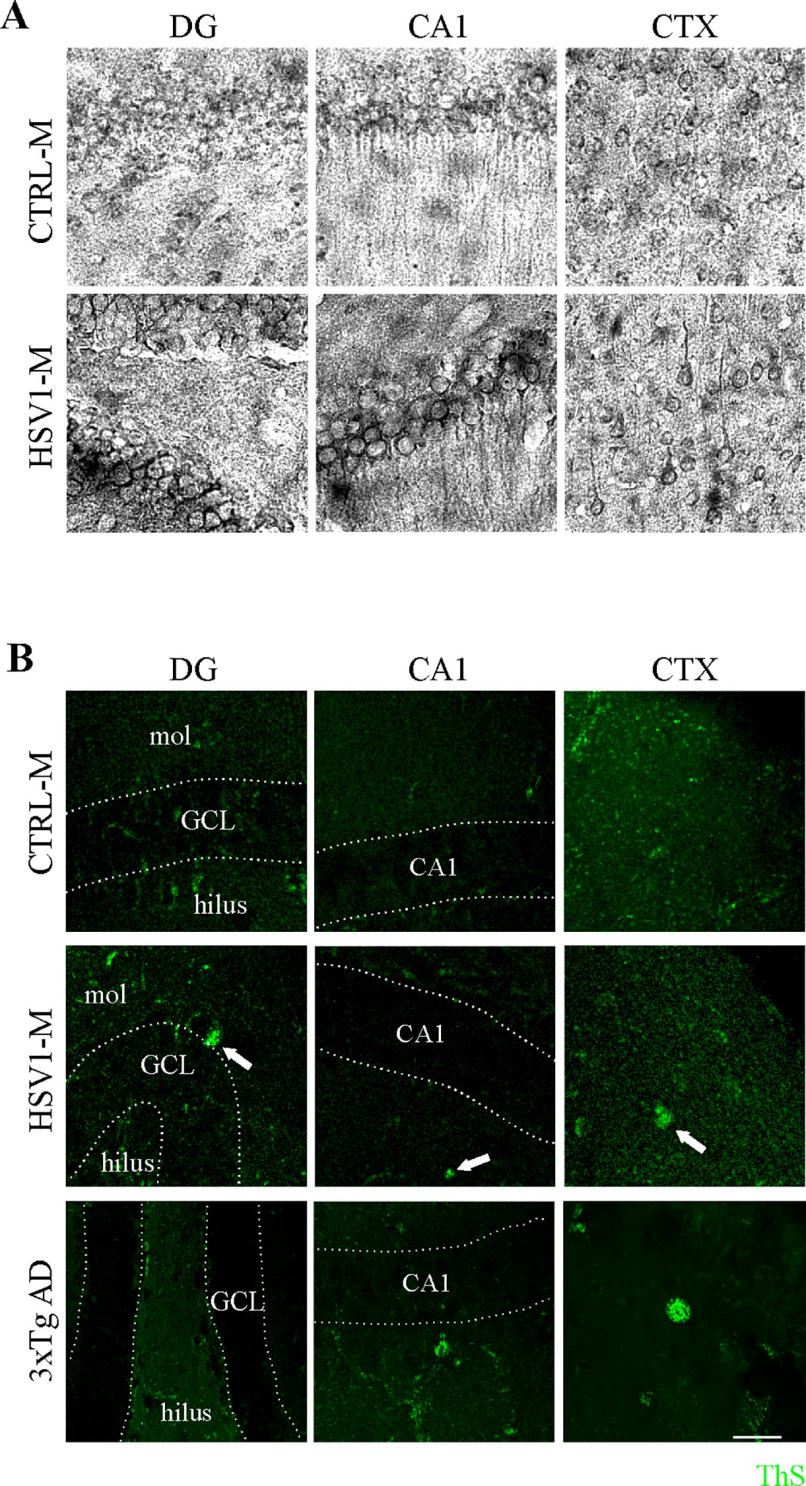
Multiple HSV-1 reactivations induce Aβ accumulation and deposition in amyloid plaques. (A) Representative images of immunoperoxidase staining of Aβ40/42 in coronal brain slices from HSV1-M and CTRL-M undergone 7 TSs. (B) Thioflavin-S (ThS) staining (green) in brain slices from mice undergone 7 TSs. Representative images of CTX, CA1 and DG are shown from 1 out of the 3 studied mice. As positive control the same analysis was performed on brain slices from 9-month-old 3×TgAD mouse. Arrowheads indicate plaques, dotted lines delimitate pyramidal neuron layer in CA1 and granule cell layer (GCL) between molecular layer (mol) and hilus in DG. Scale bars: 50 μm.

**Fig 6:**
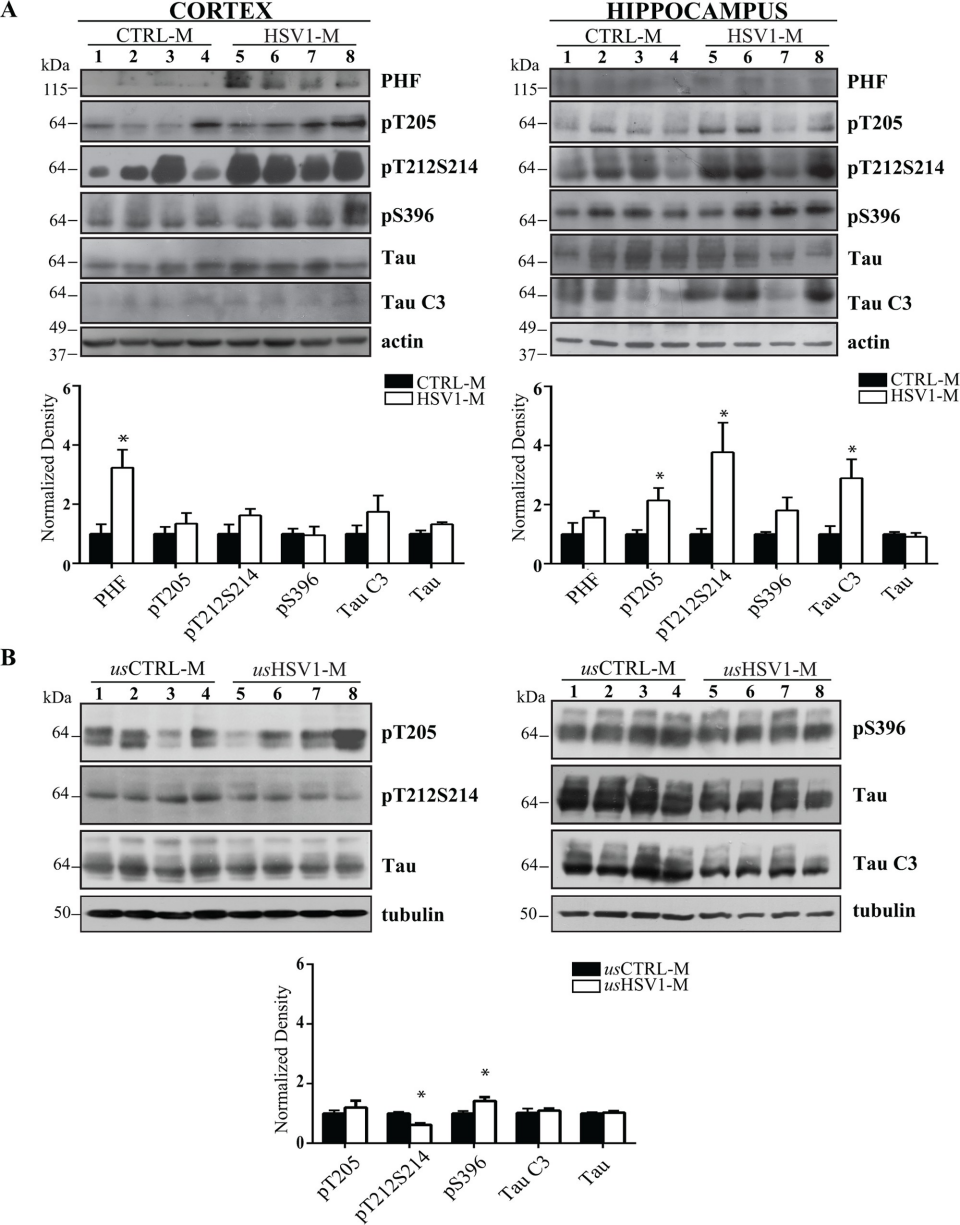
Multiple HSV-1 reactivations induce tau phosphorylation, cleavage and aggregation. Levels of phospho-tau and of its cleaved fragment TauC3 were investigated by the aid of specific antibodies in (A) neocortex (left gel) and hippocampus (right gel) homogenates from CTRL-M and HSV1-M sacrificed following 7 TSs (n = 4 for each experimental group), (B) neocortex homogenates from usCTRL-M and usHSV1-M (n = 4 for each experimental group). Actin or tubulin expression level was used as sample loading control. Densitometric analysis of immunoreactive signals normalized to matched tau (for phospho-tau) or actin (for tau [Tau] and TauC3) are shown in the graphs: values represent the normalized fold changes in protein levels from HSV1-M or usHSV1-M with respect to CTRL-M or usCTRL-M, respectively (mean ± SEM); * p<0.05 HSV1-M vs CTRL-M assessed by Mann-Whitney statistic.

**Fig 7:**
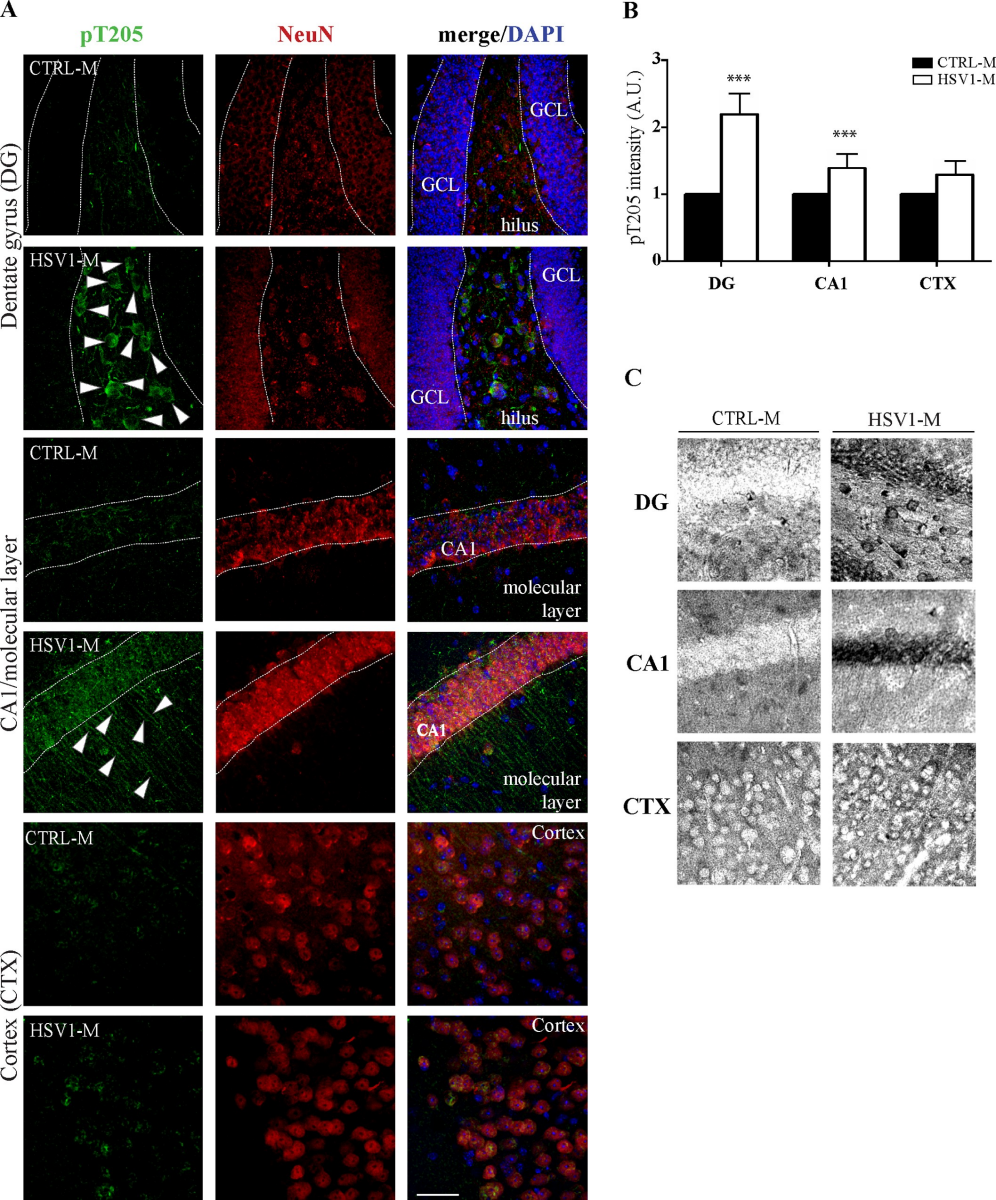
Multiple HSV-1 reactivations induce increased levels of phosphoThr205-tau (pT205). (A) Confocal immunofluorescence analysis of coronal brain slices from HSV1-M and CTRL-M undergone 7 TSs (n = 3 mice for each experimental group). Two/three coronal sections were analyzed for each brain. Phosphorylation of tau at Thr205 (pT205) was studied by a specific antibody (see S7B Fig). Neurons were identified by their immunoreactivity for anti-NeuN antibody. Cell nuclei were stained with DAPI. Panels show representative images from dentate gyrus (DG), CA1/molecular layer and somatosensory neocortex (CTX). Dotted lines delimitate pyramidal neuron layer in CA1 and granule cell layer (GCL) from hilus in DG. Arrowheads indicate pT205 in neurons of the hilus and in the axons of molecular layer. (B) Bar graphs showing pT205 fluorescence as the mean value of fluorescence quantified in DG and in CA1 of the hippocampus, and in CTX expressed as fold changes with respect to CTRL-M. Values are expressed as mean ± SEM, *** p<0.001 (p = 0.000159 for DG and CA1, p = 0.44 for CTX, assessed by ANOVA on ranks). Scale bar: 50 μm (C) Representative images of immunoperoxidase staining of pT205 in coronal brain slices from HSV1-M and CTRL-M undergone 7 TSs.

Figs 4–7 show the results from mice sacrificed after the seventh TS. Matched results from mice sacrificed after the third TS are displayed in S3 and S4 Figs.

#### Accumulation of Aβ

We first checked the presence of Aβ in mouse brains following virus reactivations. IF analysis of brain slices documented a significant accumulation of Aβ after the third TS in the dentate gyrus (DG) of hippocampus of HSV1-M respect to CTRL-M (2.4 ± 0.9 fold increase; p = 0.008, assessed by ANOVA on ranks) as well as in the CA1/molecular layer (2.3 ± 0.7; p = 0.0028) and in the somatosensory cortex (CTX; 3.0 ± 0.6, p = 0.028, S3 Fig), indicating that the virus actually induced the amyloidogenic processing of APP. Greater Aβ accumulation was found in HSV1-M undergone 7 TSs (4.6 ± 1.7 fold increase, p = 0.028 in the DG; 4.7±1.0, p = 0.0021 in the CA1; 3.0 ± 1.4, p = 0.028 in the CTX, as compared to matched CTRL-M, Fig 4). Specifically, Aβ accumulation in the DG and CA1/molecular layer was significantly greater (p = 0.037) after the seventh TS with respect to data collected after the third TS. Similar results were obtained with IPS of Aβ in brain tissues from mice undergone 7 TSs, confirming what observed by immunofluorescence (Fig 5A). Consistently, we found greater amyloid plaque deposition in HSV1-M as compared to CTRL-M, when we stained Aβ aggregates in mouse brain slices by Thioflavin-S (ThS, Fig 5B). These data suggest that HSV-1 reactivations promote accumulation of amyloidogenic APP fragments and their deposition in amyloid plaques that increased with the number of TSs.

#### Altered tau phosphorylation and cleavage

We then checked mouse brain tissue homogenates in WB for tau phosphorylation at different sites reportedly associated with neurodegeneration, i.e., threonine 205 (T205), serine 396 (S396), and double phosphorylation at threonine 212 and serine 214 (T212S214). We found a significant increase in phosphoT205-tau (pT205) and phosphoT212S214-tau (pT212S214) only in hippocampi from mice undergone 7 TSs, as compared with matched CTRL-M (p = 0.03, assessed by Mann-Whitney test, Fig 6A and S4A Fig). In neocortex samples we also found that HSV-1 multiple reactivations induced a trend increase in pT21S214 (p = 0.06) and a significant increase (p = 0.03) in a high-weight molecular band (MW>100 kDa) recognized by AT100 antibody that detects paired helical filaments (PHF)-tau, suggesting that HSV-1-induced tau hyperphosphorylation at T212S214 may promote its aggregation. On the contrary, we did not find any significant differences in phosphoS396-tau (pS396) between HSV1-M and CTRL-M.

IF staining for pT205 (Fig 7A and 7B and S4B and S4C Fig), and immunoperoxidase staining (Fig 7C), performed in brain slices following the third and the seventh reactivations, also showed a significant increase in tau phosphorylation. These data were further supported by IF staining for pT205 in brain slices from the subset of mice sacrificed before (pre-TS, n = 4) and following (post-TS, n = 3) the seventh TS (see timeline of experiments in Fig 1B): we found a significant pT205 increase in both post-TS and pre-TS HSV1-M brains as compared to CTRL-M (p≤0.001, assessed by two-way ANOVA with Tukey post-hoc correction), confirming that multiple cycles of virus replication trigger the accumulation of phosphorylated forms of tau. Interestingly, pT205 was found significantly increased also in post-TS HSV1-M brains as compared to pre-TS ones (p<0.001), indicating that virus replication induces phosphorylative events that are added to the previous, not cleared, ones (S5A and S5B Fig). Specifically, respect to pre-TS mice, post-TS HSV1-M showed a significant increase in pT205 in the DG (p = 0.041) and the CTX (p<0.001) (S5C Fig).

Furthermore, WB analysis showed that HSV-1 multiple reactivations in the brain induced a caspase-3-mediated cleavage of tau at aspartate 421, as revealed by the significant increase in TauC3 in hippocampal tissues from HSV1-M undergone 7 TSs (Fig 6A). This is considered a marker of early neurodegeneration and was previously observed both in *in vitro* and *in vivo* models of productive HSV-1 infection [26, 33–35].

Finally, to further support the role of TS-induced virus reactivation in the observed effects, we analyzed the neocortex of age-matched usCTRL-M and usHSV1-M (i.e., never undergone TS following primary infection). These tissues, that were HSV-1^+^ as demonstrated by real time PCR quantification of HSV-1 genome (85<copies/mg tissue<175, S6A Fig) revealed a different pattern of tau phosphorylation and cleavage (Fig 6B), with a significant increase (p = 0.03) in pS396 and a significant decrease in pT212S214 (p = 0.03) in usHSV1-M vs usCTRL-M, whereas no changes were found in pT205 and TauC3 levels between these UNSTRESSED experimental groups.

### Signs of neuroinflammation

**Fig 8:**
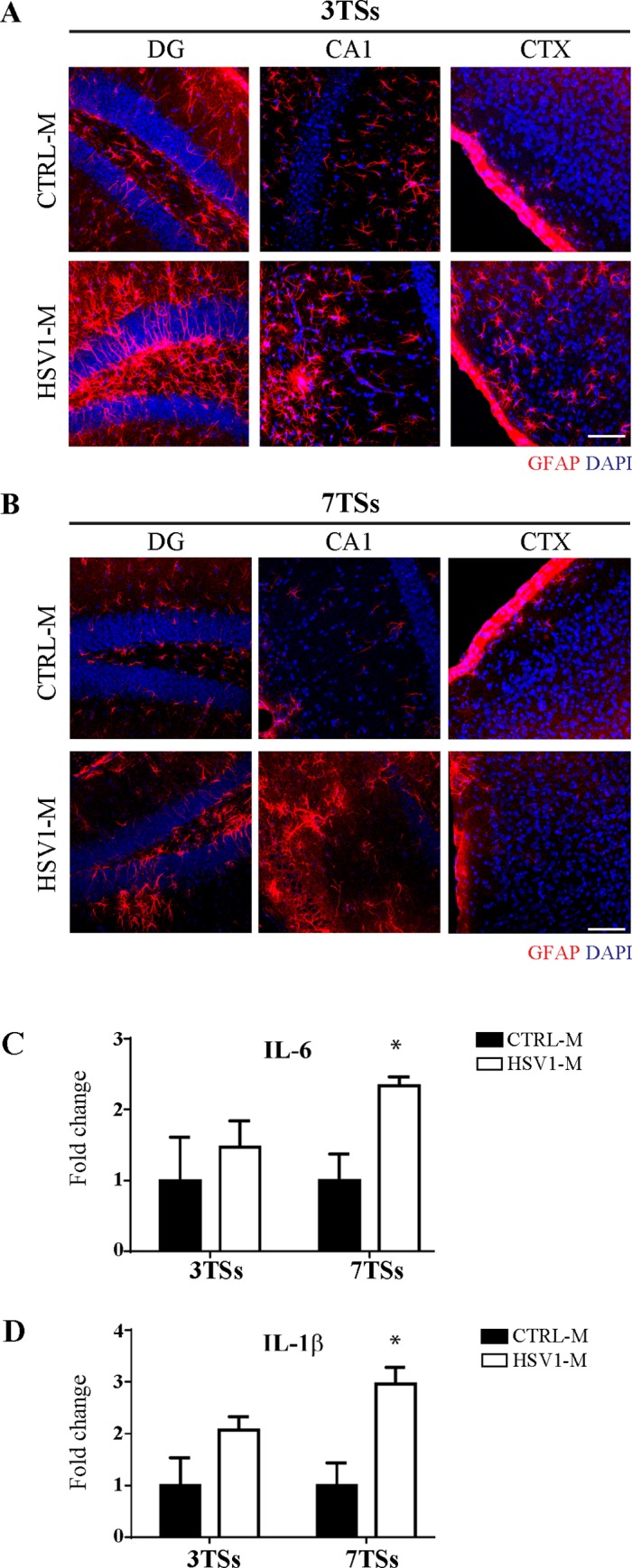
HSV-1 reactivations in the brain induce astrogliosis and increase brain levels of IL-1β and IL-6. Confocal immunofluorescence analysis of coronal brain slices from HSV1-M and CTRL-M undergone 3 TSs (A, n = 2 mice for each experimental group) and 7 TSs (B, n = 3 mice for each experimental group). Three (A) or two (B) coronal sections were analyzed for each brain. GFAP was recognized by a specific antibody (see S7B Fig). Cell nuclei were stained with DAPI. Panels show representative images from hippocampal DG and CA1, and CTX. Scale bars: 75 μm. Bar graphs showing mean IL-6 (C) and IL-1β (D) levels in cortex homogenates from HSV1-M and CTRL-M undergone 3TSs and 7TSs. Values represent the normalized fold changes in protein levels from HSV1-M with respect to CTRL-M (mean ± SEM, * p<0.05 HSV1-M *vs* matched CTRL-M).

Next, we checked whether multiple HSV-1 reactivations induced signs of neuroinflammation through IF analysis of Glial Fibrillary Acidic Protein (GFAP), a marker of astroglia, in brain slices from HSV1-M and CTRL-M. Results showed the occurrence of astrocytosis in HSV1-M brains (Fig 8A and 8B). We also measured brain levels of the proinflammatory cytokines IL-1β, which has been suggested to play a crucial role in AD pathogenesis [36–38], and IL-6, produced during HSV-1 infection [39]. Both cytokines were assessed in previously analyzed brain homogenates from mice undergone 3 and 7 TSs. Results in Fig 7C and 7D show that the cytokine levels were significantly upregulated in mice undergone 7 TSs with respect to matched CTRL-M (n = 4 for each group; p = 0.0143 for IL-6, p = 0.0107 for IL-1β, assessed by Student’s *t*-test).

### Memory loss induced by recurrent HSV-1 infections in mice

**Fig 9:**
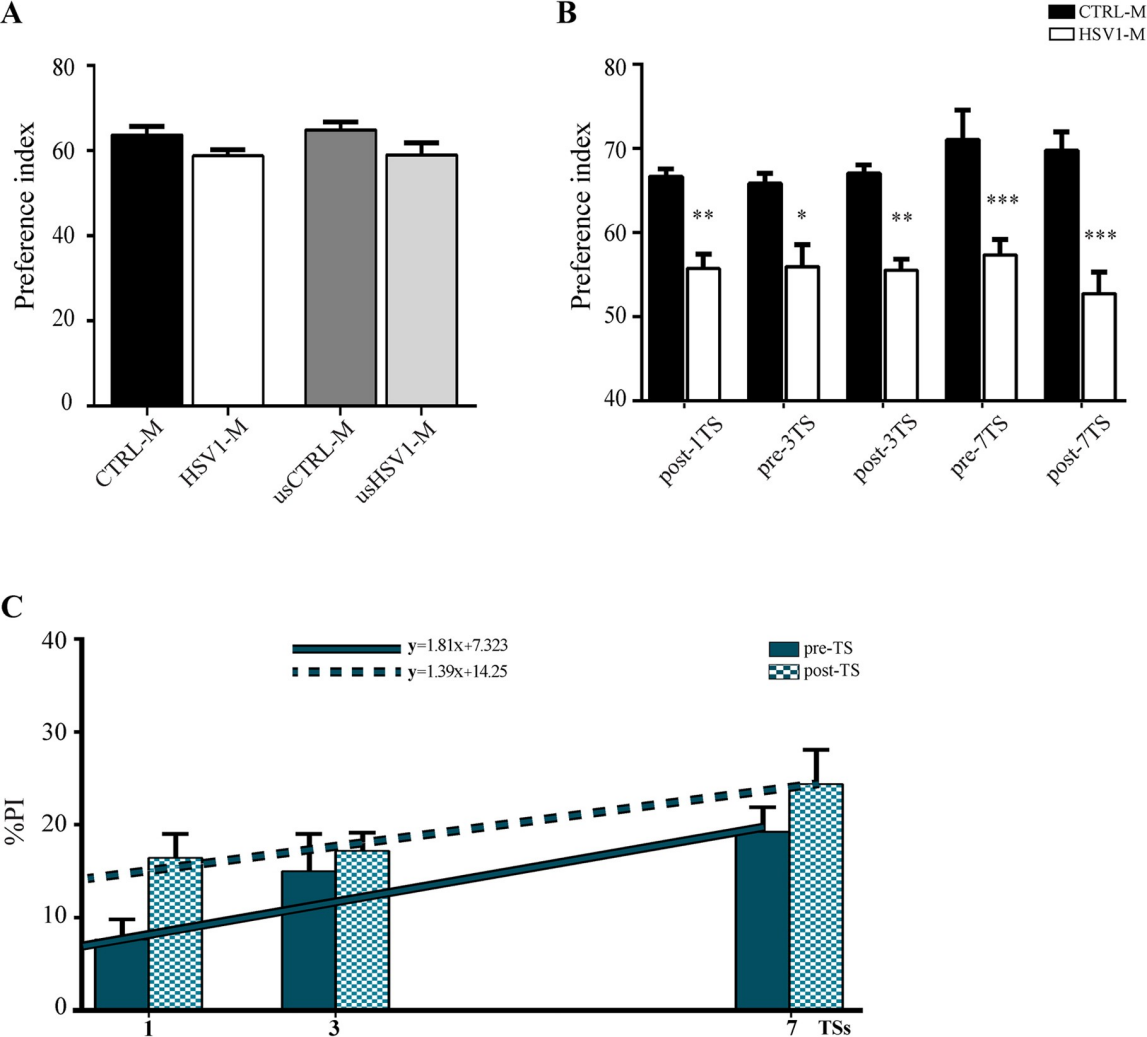
Behavioral alterations induced by recurrent HSV-1 infections in mice. (A) CTRL-M (n = 9) and HSV1-M (n = 9) were tested in NOR 5 weeks p.i.. UNSTRESSED CTRL-M (usCTRL-M, n = 6) and UNSTRESSED HSV1-M (usHSV1-M, n = 6) were tested in NOR 11 months p.i.. Bar graphs show the mean values of preference index for novel object. (B-C) NOR test was repeated one week post the 1^st^ TS (post-1TS), then one week before and after the third (pre-3TS and post-3TS, respectively) and the seventh TS (pre-7TS and post-7TS, respectively) in the same 9 CTRL-M and 9 HSV1-M tested in A, as schematized in Fig 1A. (B) Mean values of preference index for novel object are shown in the graph; * p<0.05, ** p<0.01, *** p<0.001 *vs* matched CTRL-M assessed by One WAY ANOVA with Tukey post-hoc correction; (C) % of NOR impairment in HSV1-M (*vs* matched CTRL-M) assessed as %PI (see methods) at experimental time points shown in A and B; linear regression analyses are shown as blue line for pre-TS values (F_1-25_ = 6.728, p = 0.016), and dashed blue line for post-TS values (F_1-25_ = 4.684, p = 0.04).

Finally, we checked whether accumulation of AD molecular hallmarks in HSV1-M resulted in impairment of cognitive functions. To address this issue, HSV1-M and CTRL-M were tested in Novel Object Recognition paradigm (NOR), that assesses hippocampal-dependent learning and memory. We started behavioral experiments 5 weeks after the primary infection, i.e., when latent infection was established. HSV1-M showed a slight but not significant decrease in the preference index (PI) for the novel object as compared with CTRL-M (n = 9 for both experimental groups, p = 0.0503 assessed by Mann-Whitney test, Fig 9A). When these analyses were repeated in the UNSTRESSED animals about 11 months after the primary infection, corresponding to the time point of mice tested after the seventh TS, no differences in the PI were observed between usHSV1-M and usCTRL-M (n = 6 for each experimental group, p = 0.1275 assessed by Mann-Whitney test), thus suggesting that the slight cognitive impairment induced by HSV-1 primary infection does not increase over time (Fig 9A). However, our interest was focused on the impact of repeated viral reactivations on cognitive function because of their ability to produce accumulation of AD molecular hallmarks. To this aim we assessed mouse performance in NOR one week after the first TS (post-1TS) and, subsequently, one week before (pre-) and after (post-) the third and the seventh TS (see timeline of experiments in Fig 1A). For all these tests we used the same subset of HSV1-M and CTRL-M analyzed in NOR test 5 weeks p.i.. We found that HSV1-M exhibited a significant decrease in PI as compared with CTRL-M especially after each TS (p<0.001, post-1TS; p = 0.005 post-3TS, p<0.001, post-7TS, assessed by one-way ANOVA with Tukey post-hoc correction, n = 9 for both experimental groups at each test point, Fig 9B). However, HSV1-M exhibited significant decrease in PI also when tested one week before the third and the seventh TS (p = 0.029 and p<0.001, respectively, assessed by one-way ANOVA with Tukey post-hoc correction, n = 9 for both experimental groups), suggesting that damages dependent on HSV-1 active replication may accumulate. Consistently, we also found that the cognitive deficit in HSV1-M, determined as % variation vs. matched controls (% PI, see Methods), was significantly different between pre-1TS and post-1TS (p = 0.018, assessed by Student’s *t*-test) thus supporting an association of cognitive impairment with active virus replication into the brain. In addition, %PI markedly increased with the number of virus reactivations as assessed by linear regression analysis on the pre- and post-TS values (Fig 9B and 9C, pre-TS regression: F_1-25_ = 6.728, p = 0.016; post-TS regression: F_1-25_ = 4.684, p = 0.04 for the angular coefficients). A scatterplot of individual %PI is provided in S6 Fig. These data and, in particular, those referring to the pre-TS values, that are associated to virus latency, suggest that the impairment in cognitive function in infected animals paralleled the accumulation of AD hallmarks. Indeed, those mice exhibiting phospho-tau and Aβ accumulation shown in Figs 6A and 7, also displayed impairment in terms of %PI before the seventh reactivation (the %PI being 6.1, 16.8, 35.1 and 21.2 for mice in lanes 5–8 of Fig 6A, respectively, and 18.5 for the mouse used for IF in Figs 4 and 7).

Collectively these data suggest that the HSV-1-induced cognitive impairment depends on accumulation of AD neurodegenerative hallmarks in mice brains.

## Discussion

Here, we provide novel evidence in mice that, after HSV-1 infection followed by numerous cycles of viral reactivations, the virus reaches and replicates in the brain where it triggers the progressive accumulation of AD molecular hallmarks leading to cognitive decline.

Previous studies detected signs of neurodegeneration in experimental models of encephalitis triggered by HSV-1 infection. In particular, Wozniak and colleagues showed the presence of Aβ in HSV-1-infected mouse brains 5 days after intranasal inoculation [40]. More recently, Martin and colleagues documented signs of neuroinflammation along with tau phosphorylation and cleavage in a similar model [26]. In mice injected intraperitoneally with HSV-1, Guzman-Sanchez and colleagues observed brain oxidative damage and neuroinflammation during the first month p.i. that, however, disappeared at later time points despite sporadic spontaneous viral reactivations occurred between 6 and 16 months p.i. [41].

These results are in line with the view that Aβ and tau, together with other proteins involved in neurodegeneration, play a role in the CNS innate immune response [42, 43]. In particular, Aβ antimicrobial properties against different pathogens, including HSV-1, have recently been reported [44–47]. Very recently Moir’s group reported that Aβ overexpression protects against *in vivo* and *in vitro* infections, proposing a dual protective/damaging role for this peptide [46, 48].

In our experimental model, virus infection was produced through a natural route in young mice and several virus reactivations were induced throughout their life. This allowed us to investigate the long-term effects of multiple virus reactivations commonly affecting humans over life. Remarkably, it also allowed to correlate data on viral presence/replication in the brain following TS with the progression of an AD-like phenotype.

First, we found that TS-induced HSV-1 reactivations result in the progressive accumulation of hyperphosphorylated tau and Aβ in neocortex and hippocampus, both brain regions that are mainly targeted in AD. In particular, we found Aβ localized both intraneuronally and seeded in extraneuronal ThS^+^ plaques (see Figs 4 and 5). These data are in line with previous reports of *in vitro* studies performed on HSV-1 infected neurons [19–22, 24, 49–51]. With respect to Kumar’s paper, they let also us to speculate that, in an early phase p.i, Aβ production, and likely tau phosphorylation, may concur to limit HSV-1 spread, whereas in the presence of repeated virus reactivations, Aβ accumulation may overcome a critical threshold, switching from protective to neurotoxic action. From this point of view, HSV-1, with its multiple and repeated reactivations, may represent an excellent activator for such a CNS innate response.

Aβs are peptides deriving from the APP amyloidogenic processing mediated by β- and γ-secretases. Their key role in AD, especially as oligomers, has been fully investigated [52, 53]. Accumulation of endogenous Aβ in wild type mice is quite controversial, especially their deposit in plaques. Reyes and coworkers proposed that rodents are unable to form senile plaques unless they are induced by an exogenous nucleating factor [54]. Conversely, recent papers described Aβ and plaque formation in brains from both wild type mice [55–57] and mice expressing murine APP with AD-related mutations. We also recently demonstrated that HSV-1-induced Aβ accumulation in cultured mouse cortical neurons impairs synaptic function, thus indicating that endogenous Aβ is neurotoxic [22].

More importantly, here we demonstrate that Aβ accumulation triggered by HSV-1 reactivations correlates with the cognitive impairment of infected mice (Fig 9), as also confirmed by parallel analyses of AD hallmarks accumulation in the brains of the same mice exhibiting impaired memory in behavioral tests.

Tau is a microtubule-associated protein, mainly expressed in neuronal axon, where it binds to microtubules and stabilizes their structure. Its hyperphosphorylated form loses this function, whereas it gains self-assembly properties thus forming PHFs and straight filaments subsequently aggregating in NFTs. The hyperphosphorylated forms of tau, rather than NFTs, have been shown to have a major role in behavioral impairment *in vivo* [58–60]. Our results (Fig 6) showed an increase in tau phosphorylation at T205 and S212T214, that was statistically significant in hippocampi of HSV1-M undergone 7 TSs when compared with matched CTRL-M. Consistently, IF analysis revealed a significant increment of pT205 in HSV1-M undergone multiple TSs, especially in the hilus and in CA1 regions of hippocampus, a suggested prerequisite for the induction of cognitive dysfunction [55, 59]. These phosphorylations have been reported to profoundly alter tau binding to microtubules, likely promoting protein self-assembly [61]. Consistently, we found an increased high-molecular band (MW>100 kDa), recognized by the anti-PHF-tau antibody, both in hippocampus and neocortex of HSV1-M, suggesting that the hyperphosphorylated protein aggregates in PHFs. In our model repeated virus reactivations did not cause any significant phosphorylation at S396, that was induced by acute HSV-1 infection in cultured neurons [24] and in mice [26], suggesting the induction of different phosphorylative patterns by acute or recurrent infection.

Taken together, our results suggest that the cognitive dysfunction observed in infected mice undergone several TSs is due to the action of both Aβ and tau accumulation, which may also work synergistically in producing synaptic failure. This is in line with our recent data demonstrating that Aβ and tau oligomers concur to disrupt the synaptic function in mice [62, 63].

Neuroinflammation is one of the main features of AD brains, and it may concur to AD pathogenesis [64]. Recently, Krstic and colleagues provided data supporting the idea that long-standing infection/neuroinflammation may represent a risk factor for AD [55]. According to previous studies showing the occurrence of neuroinflammation in mouse models of HSE [26], our data revealed a prominent astrogliosis following several TSs, suggesting that active HSV-1 replication in brain, that follows each reactivation, may induce neuroinflammation (Fig 8). Consistently, IL-1β and IL-6 levels in the neocortex were significantly increased after the seventh reactivation. Since most of the HSV-1-induced hallmarks are considered inflammation drivers [55, 65], our data support the hypothesis that neuroinflammation might result by both active viral replication and HSV-1-induced accumulation of neurotoxic products. In this line it is noteworthy that GFAP staining revealed astrocytosis mainly in the hilus of DG, the same hippocampal region in which we found higher levels of phospho-tau.

Finally, results from behavioral studies showed a TS-dependent impairment in HSV1-M cognitive function that started from the first TS and worsened following 7 TSs (Fig 9B and 9C). These data indicate that HSV1-M have deficits in recognition memory especially following each reactivation. Accordingly, no significant difference in PI were found between CTRL-M and HSV1-M that did not undergo TS (i.e., those tested 5 weeks p.i and the UNSTRESSED mice tested 11 months p.i., Figs 1A and 9A), suggesting that both primary HSV-1 infection and the establishment of latency are not able to induce long-lasting cognitive dysfunctions. Interestingly, HSV1-M showed a significant trend impairment in NOR performance before the seventh TS as compared with the first (Fig 9C), suggesting that damages induced by several virus reactivations may eventually accumulate and cannot be recovered. Notably, we documented that cognitive impairment in HSV1-M correlated with accumulation of AD neuropathological hallmarks. Indeed, HSV1-M also displayed significant decreases in PI before the seventh TS (i.e., when the virus should likely be latent), thus indicating that the progressive accumulation of AD hallmarks, driven by previous virus reactivation, concurred to the observed memory impairment. In this line, we found that the brain of UNSTRESSED mice exhibiting normal behavior (Fig 9A) showed a different pattern of tau phosphorylation (Fig 6B), whereas pT205 accumulation was found increased in mouse brains analyzed before the seventh TS (S5 Fig).

**Fig 10:**
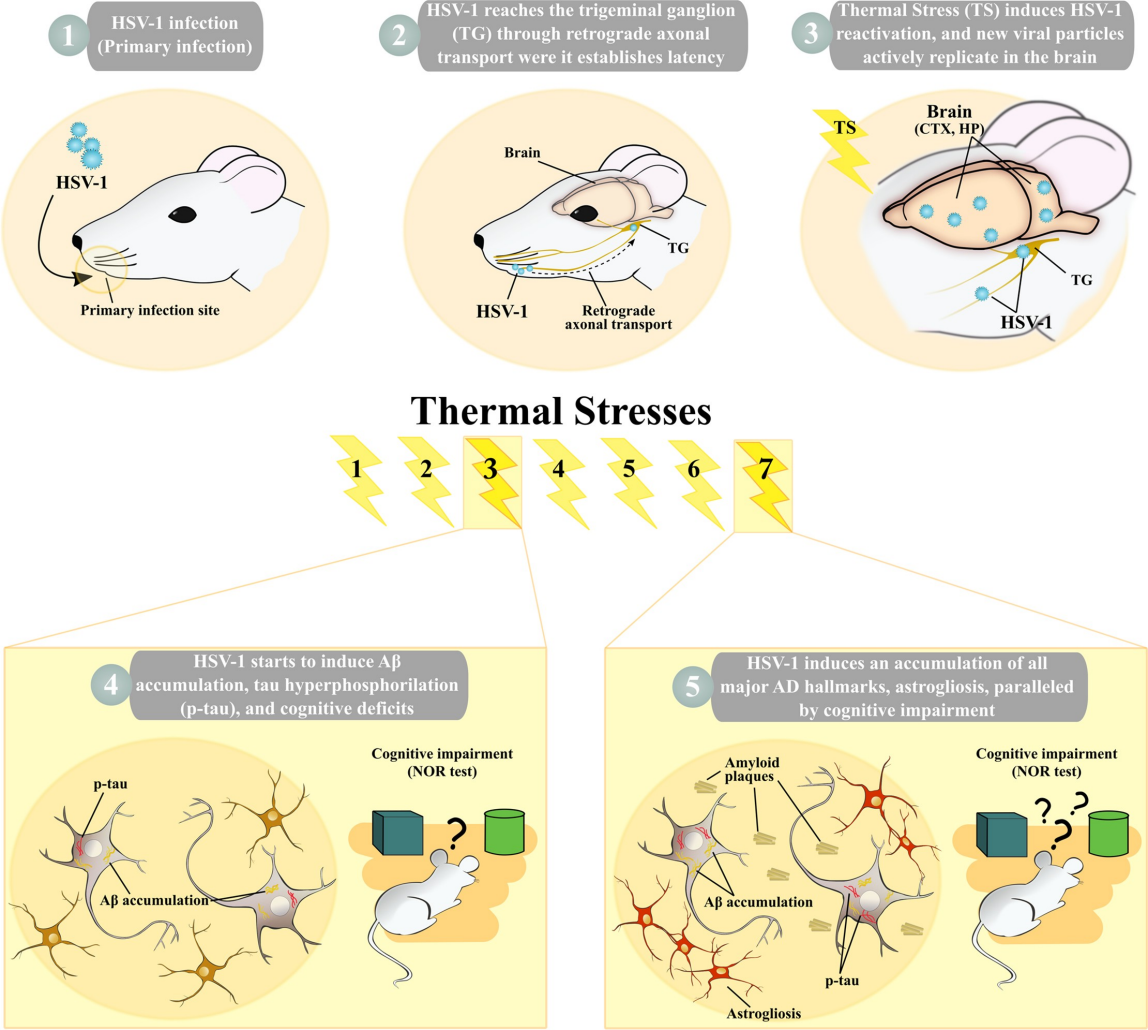
A schematic summary of the main results.

Collectively our data (summarized in Fig 10) demonstrate that damages produced by active HSV-1 replication into the brain, if repeated following multiple HSV-1 reactivations, result in an AD-like phenotype consisting in the progressive accumulation of molecular markers of neurodegeneration leading to cognitive decline. Our findings strongly support the view that recurrent HSV-1-infection is a risk factor for AD.

## Methods

### Animals and experimental groups

Six-eight-week-old female BALB/c mice (Harlan Laboratories) and 3xTg-AD mice (MMRRC, The Jackson Laboratory) were maintained under a 12 h light-dark cycle at room temperature (RT) with free access to food and water, in the animal house at Istituto Superiore di Sanità, Rome, Italy. All possible efforts were made to limit the number of animals used for experimental procedures and to minimize their suffering. One week after their arrival in animal house, mice were marked, divided into the main experimental groups (HSV-1-infected, named HSV1-M and CTRL-M, respectively) and kept in separated cages. For all the following studies we exploited 2 main cohorts of mice that were HSV-1-or mock-infected under the same experimental conditions. The first one comprised 45 HSV1-M and 29 CTRL-M: 6 of each experimental groups were separated, and tested for cognitive function 11 months p.i. and then sacrificed to analyse their brain in WB (named UNSTRESSED groups, usHSV1-M and usCTRL-M, respectively); the other mice underwent several cycles of TS as described below to induce virus reactivation (timeline of experiments in Fig 1A).

The second cohort of mice comprised 24 HSV1-M and 15 CTRL-M: 4 days p.i. 6 HSV1-M and 2 CTRL-M were sacrificed and their TGs and brain tissues analysed for the presence of the virus. The other mice, as those of the first cohort, underwent several cycles of TS as described below to induce virus reactivation and analysed as described in Fig 1B.

### Ethics statement

The authors certify that all the experimental protocols used in the present study were in compliance with the Italian and European legislation on animal experimentation (Decreto L.vo 26/2014; Direttiva n. 63/2010/EU). Animal Welfare Body of Istituto Superiore Sanità firstly reviewed experimental protocols and the Italian Ministry of Health authorized them (protocol numbers 801/2016-B, 745/2016-PR).

### Virus production and titration

HSV-1 (F strain, a wild-type strain, a kind gift from Prof. Manservigi, Ferrara University, Italy) production was performed in VERO cells, as previously reported [23]. Briefly, monolayers of kidney epithelial VERO cells (African green monkey kidney, ATCC CCL-81) were cultivated in 75 cm^2^ tissue culture flasks and infected with HSV-1 strain F at a multiplicity of infection (m.o.i.) of 0.01. After 48 h at 37°C, HSV-1-infected cells were harvested, and following 3 cycles of freeze-and-thaw, cell debris was removed with low-speed centrifugation. Similarly, mock solution consists in the supernatant of mock-infected VERO cells. In this study, the virus had a titer of 5×10^8^ PFU/ml, measured by standard plaque assay (SPA) [30].

### *In vivo* HSV-1 infection

After a slight abrasion close to the upper lip with a 21-gauge needle, HSV1-M (n = 45) and CTRL-M (n = 29) were challenged with 2 μl suspension containing 10^6^ PFU of HSV-1 or mock solution, respectively. Each treatment was done following anesthesia with intraperitoneal Ketamine (80mg/Kg) + Xylazine (5mg/Kg). All animals were daily and individually monitored for body temperature, feedings habits, weight and posture during the first week p.i. and carefully examined for the appearance of neurological signs. No death was observed following virus primary infection.

Six weeks p.i., when latent infection was established, 6 HSV1-M, and 2 CTRL-M were randomly selected and sacrificed to check the presence of the virus in the brain by PCR amplification of viral TK gene (see below).

To perform further experiments, 24 mice were HSV-1-infected (HSV1-M) and 15 mice were mock-infected (CTRL-M) under the same experimental condition described before and used for the following analyses: a) to assess the efficiency of virus inoculation, 6 randomly selected HSV1-M and 2 CTRL-M were sacrificed 4 days p.i.. TGs were then collected and individually processed for virological tests (2 TGs for each test, see below) and for WB analysis for ICP4 (6 TGs for HSV-M and 2 for CTRL-M). Further virological analyses were performed with theirs tissue homogenates. As most of the mice from the first round of infection, these animals undergo several cycles of TS to induce virus reactivation and were analyzed as described below (see timeline of experiments in Fig 1B).

### HSV-1 reactivation

Six weeks p.i., 51 HSV1-M (33 and 18 belonging to the first and the second cohort, respectively) and 34 CTRL-M (21 and 13 belonging to the first and the second cohort, respectively) were individually subject to TS to reactivate latent virus, according to reported protocols [27] with minor modifications. Briefly, mice were kept for 15 min into restrainers suspended in a constant-temperature circulating water bath (set at 43°C), in order to maintain their body temperature at 40–42°C, and then placed under a warm lamp for 30 min to prevent hypothermia. Body temperature was continuously monitored prior, during (at 3–4 min of interval) and after TS by a flexible rectal temperature probe (Acorn Temp JKT Thermocouple Thermometer, Kent Scientific Corp). TS was repeated up to seven times at 6–8 weeks of interval. Low mortality rate in mice (8–12%) within the 11-month protocol schedule was observed. Mice were analyzed at specific checkpoints as described below and in Fig 1. To detect the presence of virus in the brain, some animals (see figures and relative legends for n in each group, time point and analysis) were sacrificed 24 h post the first, third and seventh TS, their brains were collected, and analyzed for TK gene, ICP4 mRNA or gB, as described below. A subset of 4 mice for HSV1-M and CTRL-M was sacrificed 24 h post the third TS, TGs and different brain areas were collected and analyzed for the presence of infectious virus. Another subset of mice was sacrificed before (n = 7 HSV1-M, n = 4 CTRL-M) or after (n = 5 HSV1-M, n = 3 CTRL-M) the seventh TS. Some of them were perfused and analyzed in IF for the presence of gB or pTau205; TGs and different brain areas were collected from not perfused mice and analyzed for the presence of infectious virus.

### Virus titration in TGs and in Brains

To isolate infectious virus, tissues were treated as follows:

1. TGs were collected, homogenized in 10% heat-inactivated fetal bovine serum (FBS) in RPMI supplemented with 1% glutamine, 100 U/ml penicillin, and 100 μg/ml streptomycin (complete-RPMI);
2. Brain areas collected from mice sacrificed before and 24 h after TS were incubated in serum-free RPMI containing 0.08% trypsin, 0.013% collagenases type-IA (Sigma-Aldrich), penicillin and streptomycin, as described [66]. After 40 min at 37°C, the suspension was centrifuged at 4°C, the pellet re-suspended in complete-RPMI.

Infectious virus was then titrated with the following procedures:

- After three freeze-thawing cycles, tissue homogenates from 1 and 2 were serially diluted in RPMI and incubated on confluent monolayers of VERO cells. After 3 h at 37°C, supernatants were discarded, cells were washed with PBS (Sigma-Aldrich), and incubated for 4 days with 5% FBS-RPMI, 1% glutamine, 100 U/ml penicillin, and 100 μg/ml streptomycin, at 37°C in 5% CO_2_. Cell supernatants were then harvested and analyzed for the presence of infectious virus on fresh monolayers of VERO cells by a) SPA (performed with 1.5% carboximethylcellulose), b) Reed and Muench method (TCID_50_) [31], c) ICW assay for evaluation of viral gB protein expression [32]. For b) and c), dilutions of supernatants were incubated on cells seeded on 96-well plate. Cytopathic effect (b) or gB expression (c) was evaluated 3 days later. Parallel ICW assay was performed on VERO cells infected for 24 h with serial dilutions of stock HSV-1 as previously described [32].
- After three freeze-thawing cycles tissue homogenates from 1 and 2 were serially diluted in RPMI and incubated on confluent monolayers of VERO cells. After 3h at 37°C, supernatants were discarded, and cells washed with PBS and infectious virus detected by ICW. In parallel, ICW assay was performed on VERO cells infected for 24 h with serial dilutions of stock HSV-1 to generate a standard curve as previously described [32].

TCID_50_ conversion to PFU/ml was performed as described [67] by the following formula:

PFU/ml/TCID_50_/ml = 0.7

### PCR

TGs and brain tissues (as whole encephalon or isolated Cortex, hippocampus and cerebellum) were removed from mice, weighed, frozen, and stored at −80°C until use. Total RNA and DNA were extracted from mouse TG or brain tissues (10–20 and 20–30 mg, respectively) by using AllPrep DNA/RNA/Protein Mini 406 Kit (Qiagen), according to manufacturer’s protocols.

Qualitative PCR assay: the isolated RNA was treated with DNase I (Invitrogen, Life Technologies, Monza, Italy). The RNA/DNA quality and quantity were verified spectrophotometrically (Pearl Nanophotometer, IMPLEN). 1 μg of RNA was used as a template for generating cDNA (iScript cDNA Synthesis Kit, Bio-Rad), the complete reaction mix was incubated in a thermal cycler using the following protocol: Priming 5 min at 25°C, Reverse transcription 20 min at 46°C, RT inactivation 1 min at 95°C.

Five μl cDNA was used for the amplification of ICP4, 1 μg DNA was used for amplification of Tk, and β-actin gene was used as an endogenous control for the PCR reaction. PCR was performed with the iTaq DNA Polymerase cod. 1708870 (Bio-Rad) on an iQ5 Real-Time PCR Detection system (Bio-Rad), according to the manufacturer’s recommendations. The PCR reaction was performed with the corresponding PCR primers (S7A Fig), which ensured the specificity of the PCR products. PCR cycles were as follows: 94°C for 5 min, followed by 35 cycles of 94°C for 30 s, 58°C for 30 s, and 72°C for 30 s.). PCR products were separated in 1% agarose gels.

Quantitative real-time PCR assay (qPCR): the number of viral glycoprotein D (gD, US6 region) copies was determined by qPCR using the HSV1 ELITe MGB Kit according to manufacturer’s protocols. A same aliquot of extracted DNA for each sample were subjected to 45 cycles of PCR amplification (94°C, 10 s; 60°C, 30 s; 72°C 20s) using 7300 Real-Time PCR System. HSV-1 viral load levels were then normalized and expressed as the number of HSV-1 DNA copies per mg of tissue.

### Behavioral tests

A set of animals was tested in the NOR paradigm to investigate hippocampal-dependent learning and memory [68]. The NOR protocol, lasting three consecutive days, was performed as previously described [69]. On the first day (familiarization phase), each mouse was allowed to freely explore the empty arena (45×45cm) for 10 min. On the second day (training phase), each mouse was placed for 10 min in the arena containing two identical objects in a symmetric position from the center: an explorative behavior was scored when the head of the animal was facing close (< 2 cm away) to the object or any part of the body, except the tail, was touching the object. The time spent exploring each object was recorded. The animals were returned to their home cages immediately after training. On the third day (test phase), a novel object replaced one of the familiar objects used during the training and the animals were allowed to explore freely for 10 min. During this session we measured the animal “preference index” (PI) for a novel object, expressed as the ratio between the novel object exploration time and the total exploration time.

All objects were balanced in terms of physical complexity and were emotionally neutral. The arena and the objects were cleaned by 70% alcohol after each session to avoid possible odorant cues.

To determine whether memory alteration in HSV1-M *vs*. CTRL-M worsened with the number of TSs, we measured the “percent variation” in preference index (%PI) between the PIs found in individual HSV1-M before or after each TS and the mean values of PI obtained for all CTRL-M for each TS as follows:

$$\%PI{\left(HSV1-M\right)}_{X}=100-\left(\frac{PI{\left(HSV1-M\right)}_{X}}{\frac{\sum_{Y=1}^{N}{PI(CTRL-M)}_{Y}}{N}}*100\right)$$

If no memory alterations are present %PI is, by definition, equal to 0.

### Immunofluorescence and immunohistochemistry

Mice were anesthetized by intraperitoneal injection of a cocktail of Ketamine (200 mg/Kg), Xylazine (10 mg/Kg) and transcardially perfused with PBS (pH 7.4), followed by 4% paraformaldehyde (PFA) in PBS. The brains were post-fixed for 24 h at 4°C, followed by cryoprotection for 24 h in 15%, and then, in 30% sucrose in PBS. Coronal slices (40-μm thick) containing the hippocampi were cut with a vibratome (VT1000 S, Leica Microsystems). Serial sections (one of 6, spacing 240 μm) were collected in order to cover all hippocampal formation and stored at -20°C in cryoprotectant solution (Sigma-Aldrich) until immunohistochemical evaluation. Matched slices (e.g., the first and the fourth or the first, the third and the sixth of every series) were then washed with PBS, permeabilized with 0.5% Triton X-100 in PBS (15 min, RT), incubated (90 min) with 10% horse serum (HS) and 0.2% Triton X-100 in PBS to block unspecific binding sites, and then incubated overnight at 4°C with specific dilution of primary antibodies in 5% HS, 0.2% Triton X-100 in PBS (listed in S7B Fig). Following 3×10 min washes in PBS, free-floating slices were incubated with specific secondary antibodies coupled to Alexa Fluor 488 or Alexa Fluor 546 dyes (1:1000, Thermo Fisher; 90 min at RT). The specificity of antibody staining was checked by performing control staining on random selected slices only with secondary antibodies. Following 3×10 min PBS washes, slices were incubated with 4’,6’-Diamidine-2’-Phenylindole Dihydrochloride (DAPI, 1:500 dilution in PBS, Thermo; 20 min at RT), washed, mounted on coverslip with ProLong Gold Anti-fade reagent (Thermo) and air-dried in the dark. Diaminobenzidine (DAB, Impact DAB, Vector Laboratories) detection of immunohistochemical signals was performed following quenching of endogenous peroxidase activity with 0.3% H_2_O_2_ in PBS/MeOH (1:1; 15 min at RT). Vectastain Elite-ABC Kit (Vector Laboratories) was then used according to manufacturer’s instructions, and slices were stained for 5–10 minutes.

For detection of Aβ aggregate deposition, following the permeabilization step, slices were incubated (8 min at RT) with 0.5% Thioflavin S (ThS) solution (Sigma-Aldrich) in 50% filtered MeOH, followed by 2×5 min washes in 70% MeOH, 1×5 min wash in 0.1 M phosphate buffer and 1×15 min wash in PBS. Brain sections were then mounted with ProLong Gold Anti-fade reagent and air-dried in the dark. As positive control, the same analysis was performed on coronal brain slices from 9-month-old 3×Tg-AD mice [70].

Twenty micron-thick confocal stacks made of images (1024×1024 pixels) were acquired at 40× or 60× magnification with a confocal laser scanning system (Nikon A1 MP) and an oil-immersion objective (N.A. 1.4). For some images, additional 3× or 4× magnification was applied. Fluorescent dyes were excited at 488 and 546 nm with diode lasers. Quantification of immunofluorescence was carried out in the maximum projection images of the acquired confocal stacks by drawing regions of interest (ROIs) in specific brain areas of the slices (CTX, DG and CA1 regions of the hippocampus) and quantifying the mean fluorescence intensity in the ROIs.

### Western blotting

Mice were decapitated, and then the hippocampi, cortices or TGs were immediately dissected and stored into liquid nitrogen and then kept at -80°C until use. Tissues were homogenized on ice, in 5 volumes of RIPA buffer (20 mM Tris, 150 mM NaCl, 1% Triton X-100, 1% sodium deoxycholate, 0.1% SDS) containing protease and phosphatase inhibitors (Sigma-Aldrich), and separated by centrifugation at 15000×g at 4°C for 20 min. Total protein concentrations were measured with Micro BCA method (Thermo Fisher Scientific). Protein samples (20–30 μg) were separated by 7.6–15% SDS-PAGE, blotted onto 0.45 μm nitrocellulose (Amersham Protran 0.45 μm, GE Healthcare). For phospho-tau detection, after blotting, the nitrocellulose membranes were dried at RT (20 min), boiled in PBS and rehydrated in Tris buffer saline (TBS). Membranes were blocked in 10% not-fat milk (3–5 h), in 0.1% Tween-20 TBS (T-TBS) and incubated overnight at 4°C with the primary antibody dilution in 5% not-fat milk in T-TBS. They were then washed (4×10 min washes) with T-TBS and incubated with secondary antibody (horseradish peroxidase-conjugated antibodies, Jackson ImmunoResearch Laboratories). List of primary antibodies/dilution is shown in S7B Fig. Following 4×10 min washes in T-TBS, immunosignals were detected by the enhanced chemiluminescence reaction (Amersham Biosciences). Densitometric analysis was performed using Quantity One software (Bio-Rad): the integrated density, corrected for non-specific background and equal loading/native protein (β-Actin or -tubulin/Tau), were averaged per animal and included in the statistical analysis.

### Cytokine detection

Elisa kits (R&D systems) were used to assess cytokines IL-1ß and IL-6 levels in brain homogenates from CTRL-M and HSV1-M undergone 3 and 7 TSs, according to the manufacturer’s instructions.

### Statistics

Statistical comparisons were performed with GraphPad 6.0 (Prism) and Sigmaplot 14.0 (Systat software Inc.) by using one-way ANOVA or ANOVA on ranks, linear regression analysis, Student’s *t*-test, or Mann-Whitney test, when appropriate. Data are presented as means ± Standard Deviation (SD) or as mean ± standard error of the mean (SEM) when appropriate. The level of significance was set at 0.05. In all experiments the operators were blind to the study conditions.

## Supporting information

S1 Fig Animal body weight and virus replication in mouse brains. (A) Body weight assessed for HSV1-M (n ranging from 29 to 9) and CTRL-M (n ranging from 19 to 2), expressed as mean grams (g) from mice weighted during the experimental protocol from 2 to 13 months of age, in function of age (months); red lines indicate the time points of primary infection and selected TSs. (B) Representative image of RT-PCR amplification of ICP4 mRNA in 3 out of 18 HSV1-M TK^+^ brains and CTRL-M brain. C = negative control of RT-PCR assay. The analyses were performed on subsets of mice sacrificed following the 1^st^, 3^rd^, and 7^th^ TS. V = HSV-1 ICP4 amplification as positive control. The number of studied brains and the percentages of ICP4^+^ brains are shown under the gel. (C) Confocal immunofluorescence analysis of gB and Glial Fibrillary Acidic Protein (GFAP) expression in coronal brain slices from HSV1-M (n = 2) undergone 3TSs. Cell nuclei were stained with DAPI. Two/three randomly selected coronal sections were analyzed for each brain. Panels show representative images of dentate gyrus (DG, upper) and molecular layer of the hippocampus (middle) and somatosensory cortex (lower). Scale bar: 50 μm. Arrowheads indicate gB^+^ cells.
(TIF)

S2 Fig Efficacy of TS in inducing virus reactivation. (A) Confocal immunofluorescence analysis of gB and GFAP expression in coronal brain slices from 4 HSV1-M and 2 CTRL-M undergone 6 TSs and sacrificed before the 7^th^ TS (pre-TS) and from 3 HSV1-M and 1 CTRL-M sacrificed following the 7^th^ TS (post-TS). Two coronal sections were analyzed for each brain. Cell nuclei were stained with DAPI. Panels show representative images from the DG. Insets show higher magnification (3×) of boxes outlined in each panel. Dotted lines delimitate pyramidal neuron layer in CA1 and granule cell layer (GCL) from hilus in DG. Scale bar: 50 μm. (B) TG and the indicated brain tissues were harvested from 3 HSV1-M and 2 CTRL-M sacrificed before the 7^th^ TS (pre-TS) and 2 HSV1-M and 1 CTRL-M sacrificed 24 h post the 7^th^ TS (post-TS), homogenized as described in Methods, and directly tested on VERO cells by ICW assay for assessing the presence and the titer of infectious virus. Bar graph shows the mean values ± SEM of virus titer expressed as log_10_ PFU/ml.
(TIF)

S3 Fig Aβ levels in brain slices from mice sacrificed following 3TSs. Confocal immunofluorescence analysis of coronal brain slices from HSV1-M and CTRL-M undergone 3 TSs (n = 2 mice for each experimental group). Two/three coronal sections were analyzed for each brain. Aβ40/42 were recognized by immunoreactivity for a specific antibody (see S7B Fig). Neurons were identified by their immunoreactivity for anti-NeuN antibody. Cell nuclei were stained with DAPI. Panels show representative images from the DG, CA1 and somatosensory neocortex (CTX). Insets show higher magnification (3×) of boxes outlined in each panel. Dotted lines delimitate pyramidal neuron layer in CA1 and granule cell layer (GCL) from hilus in DG. Scale bar: 50 μm. Bar graphs showing mean Aβ fluorescence intensity quantified in the studied brain areas and expressed as fold change with respect to CTRL-M. Data are represented as mean ± SEM, * p<0.05, ** p<0.01.
(TIF)

S4 Fig Pattern of tau phosphorylation and cleavage in brain homogenates from mice undergone 3 TSs. (A) Levels of phospho-tau and of its cleaved fragment TauC3 were investigated by using specific antibodies (see S7B Fig) in neocortex (upper gels) and hippocampus (bottom gels) homogenates from CTRL-M and HSV1-M sacrificed following 3 TSs (n = 3 for each experimental group). Actin expression level was used as sample loading control. Densitometric analysis of immunoreactive signals normalized to tau (for phospho-tau) or actin (for tau [Tau] and TauC3) are shown in the bar graphs: values represent the normalized fold changes in protein levels from HSV1-M with respect to CTRL-M (mean ± SEM, n = 3); * p<0.05 HSV1-M vs CTRL-M. (B) Confocal immunofluorescence analysis of coronal brain slices from HSV1-M and CTRL-M undergone 3 TSs (n = 2 mice for each experimental group). Two/three coronal sections were analyzed for each brain. Phosphorylation of tau at Thr205 (pT205) was studied by a specific antibody (see S7B Fig). Neurons were identified by their immunoreactivity for anti-NeuN antibody. Cell nuclei were stained with DAPI. Panels show representative images from dentate gyrus (DG), CA1 and somatosensory neocortex (CTX). Dotted lines delimitate pyramidal neuron layer in CA1 and granule cell layer (GCL) from hilus in DG. Arrowheads indicate pT205 in neurons of the hilus and in the axons of molecular layer. Scale bar: 50 μm. (C) Bar graphs showing pT205 fluorescence as the mean value of fluorescence quantified in DG and CA1 areas of the hippocampus and neocortex, expressed as fold changes with respect to CTRL-M. Values are expressed as mean ± SEM, *** p<0.001 (p = 0.000583 for DG and CA1, p = 0.20 for CTX, assessed by ANOVA on ranks).
(TIF)

S5 Fig Multiple HSV-1 reactivations induce accumulation of pT205. (A) Confocal immunofluorescence analysis on coronal brain slices from 4 HSV1-M and 2 CTRL-M undergone 6 TSs and sacrificed before the 7^th^ TS (pre-TS) and from 3 HSV1-M and 1 CTRL-M sacrificed following the 7^th^ TS (post-TS). Phosphorylation of tau at Thr205 (pT205) was studied by a specific antibody (see S7B Fig). Neurons were identified by their immunoreactivity for anti-NeuN antibody. Cell nuclei were stained with DAPI. Panels show representative images from dentate gyrus (DG), CA1/molecular layer and somatosensory neocortex (CTX). Dotted lines delimitate pyramidal neuron layer in CA1 and granule cell layer (GCL) from hilus in DG. Arrowheads indicate pT205 in neurons of the hilus and in CA1. (B) Bar graphs show pT205 intensity as the mean value of fluorescence quantified in the analyzed slices and expressed as fold changes with respect to CTRL-M. Values are expressed as mean ± SEM, *** p<0.001, ** p<0.01 (p<0.001 for post-TS vs pre-TS and vs CTRL-M; p = 0.001 for pre-TS vs CTRL-M, assessed by two-way ANOVA with Tukey post-hoc correction). (C) Bar graphs show pT205 intensity as the mean value of fluorescence quantified in the indicated brain areas and expressed as fold changes with respect to CTRL-M. Values are expressed as mean ± SEM, *** p<0.001, * p<0.05 *vs* pre-TS (p = 0.041 for DG and p<0.001 for CTX)
(TIF)

S6 Fig (A) Scatter dot plot showing qPCR quantification of the HSV-1 copies/mg in cortex homogenates from the 4 usHSV1-M analyzed in WB (see Fig 6B). Matched usCTRL-M were assessed as control. (B) Scatterplots showing individual %PI values of 9 HSV1-M *vs* matched CTRL-M tested in NOR one week before (left graph, pre-TS values) and one week after (right graph, post-TS values) the 1^st^ and 7^th^ TS.
(TIF)

S7 Fig (A) Table summarizing the specific primers used for PCR detection of viral TK and cellular actin DNA, as well as for ICP4 and actin mRNA. (B) Table summarizing antibodies and their dilutions used for immunoblotting (WB), immunofluorescence (IF) or immunoperoxidase staining (IPS).
(TIF)
