# Supplementary figures and images for: Recurrent herpes simplex virus-1 infection induces hallmarks of neurodegeneration and cognitive deficits in mice
Source: PLoS Pathog. 2019 Mar 14;15(3):e1007617. doi: 10.1371/journal.ppat.1007617 (PMC6417650; doi:10.1371/journal.ppat.1007617)

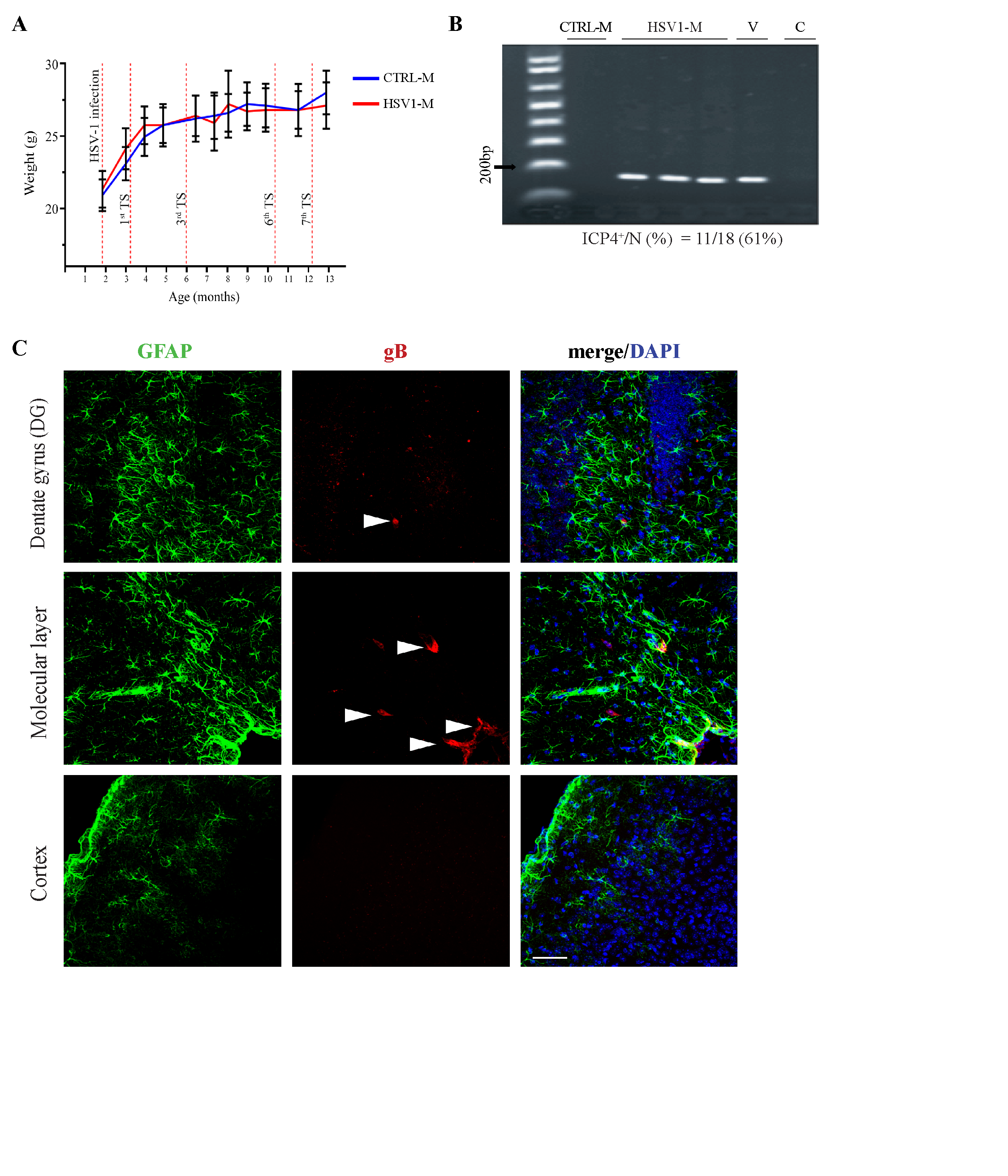

Supplement: S1 Fig — (A) Body weight assessed for HSV1-M (n ranging from 29 to 9) and CTRL-M (n ranging from 19 to 2), expressed as mean grams (g) from mice weighted during the experimental protocol from 2 to 13 months of age, in function of age (months); red lines indicate the time points of primary infection and selected TSs. (B) Representative image of RT-PCR amplification of ICP4 mRNA in 3 out of 18 HSV1-M TK+ brains and CTRL-M brain. C = negative control of RT-PCR assay. The analyses were performed on subsets of mice sacrificed following the 1st, 3rd, and 7th TS. V = HSV-1 ICP4 amplification as positive control. The number of studied brains and the percentages of ICP4+ brains are shown under the gel. (C) Confocal immunofluorescence analysis of gB and Glial Fibrillary Acidic Protein (GFAP) expression in coronal brain slices from HSV1-M (n = 2) undergone 3TSs. Cell nuclei were stained with DAPI. Two/three randomly selected coronal sections were analyzed for each brain. Panels show representative images of dentate gyrus (DG, upper) and molecular layer of the hippocampus (middle) and somatosensory cortex (lower). Scale bar: 50 μm. Arrowheads indicate gB+ cells. (TIF) [file ppat.1007617.s001.tif]

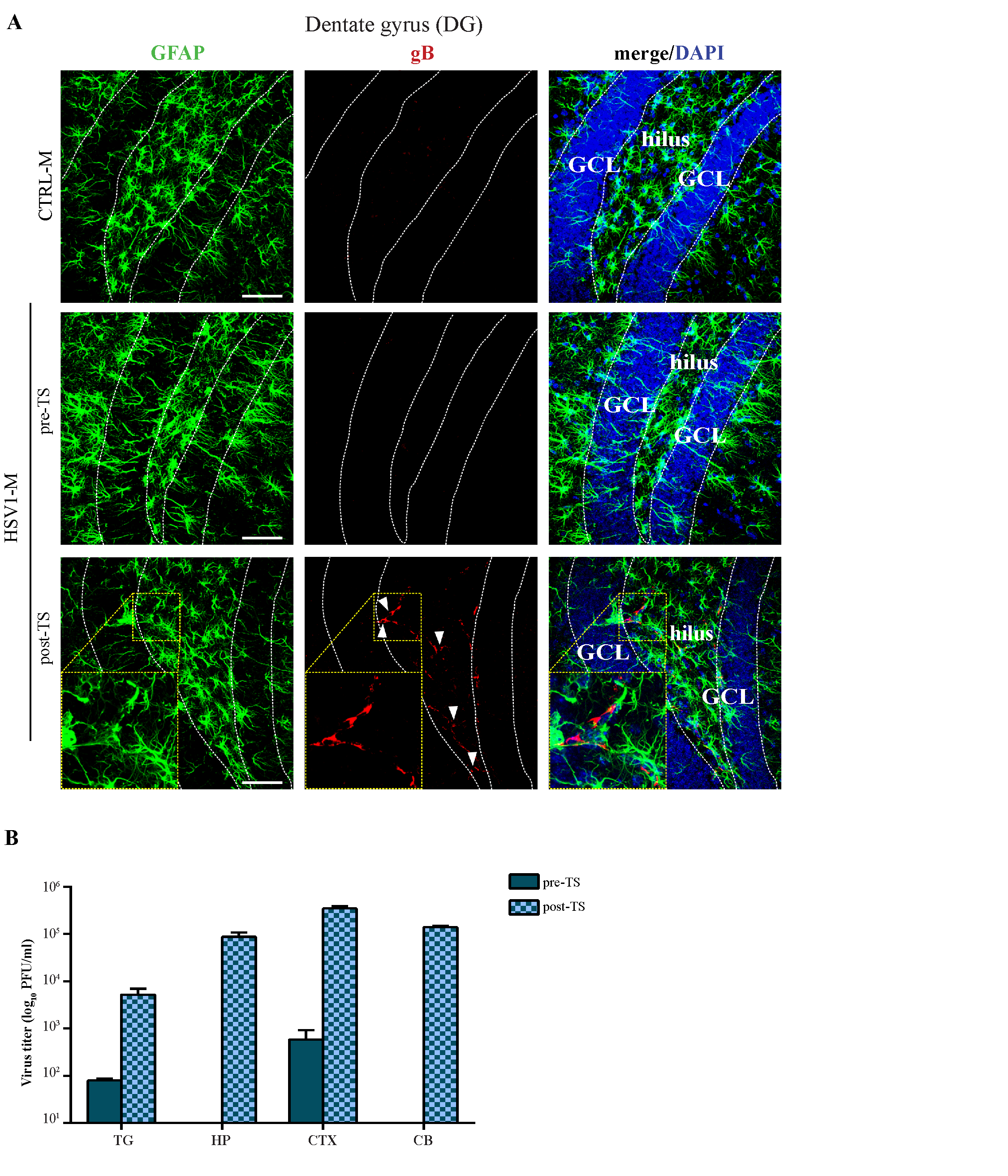

Supplement: S2 Fig — (A) Confocal immunofluorescence analysis of gB and GFAP expression in coronal brain slices from 4 HSV1-M and 2 CTRL-M undergone 6 TSs and sacrificed before the 7th TS (pre-TS) and from 3 HSV1-M and 1 CTRL-M sacrificed following the 7th TS (post-TS). Two coronal sections were analyzed for each brain. Cell nuclei were stained with DAPI. Panels show representative images from the DG. Insets show higher magnification (3×) of boxes outlined in each panel. Dotted lines delimitate pyramidal neuron layer in CA1 and granule cell layer (GCL) from hilus in DG. Scale bar: 50 μm. (B) TG and the indicated brain tissues were harvested from 3 HSV1-M and 2 CTRL-M sacrificed before the 7th TS (pre-TS) and 2 HSV1-M and 1 CTRL-M sacrificed 24 h post the 7th TS (post-TS), homogenized as described in Methods, and directly tested on VERO cells by ICW assay for assessing the presence and the titer of infectious virus. Bar graph shows the mean values ± SEM of virus titer expressed as log10 PFU/ml. (TIF) [file ppat.1007617.s002.tif]

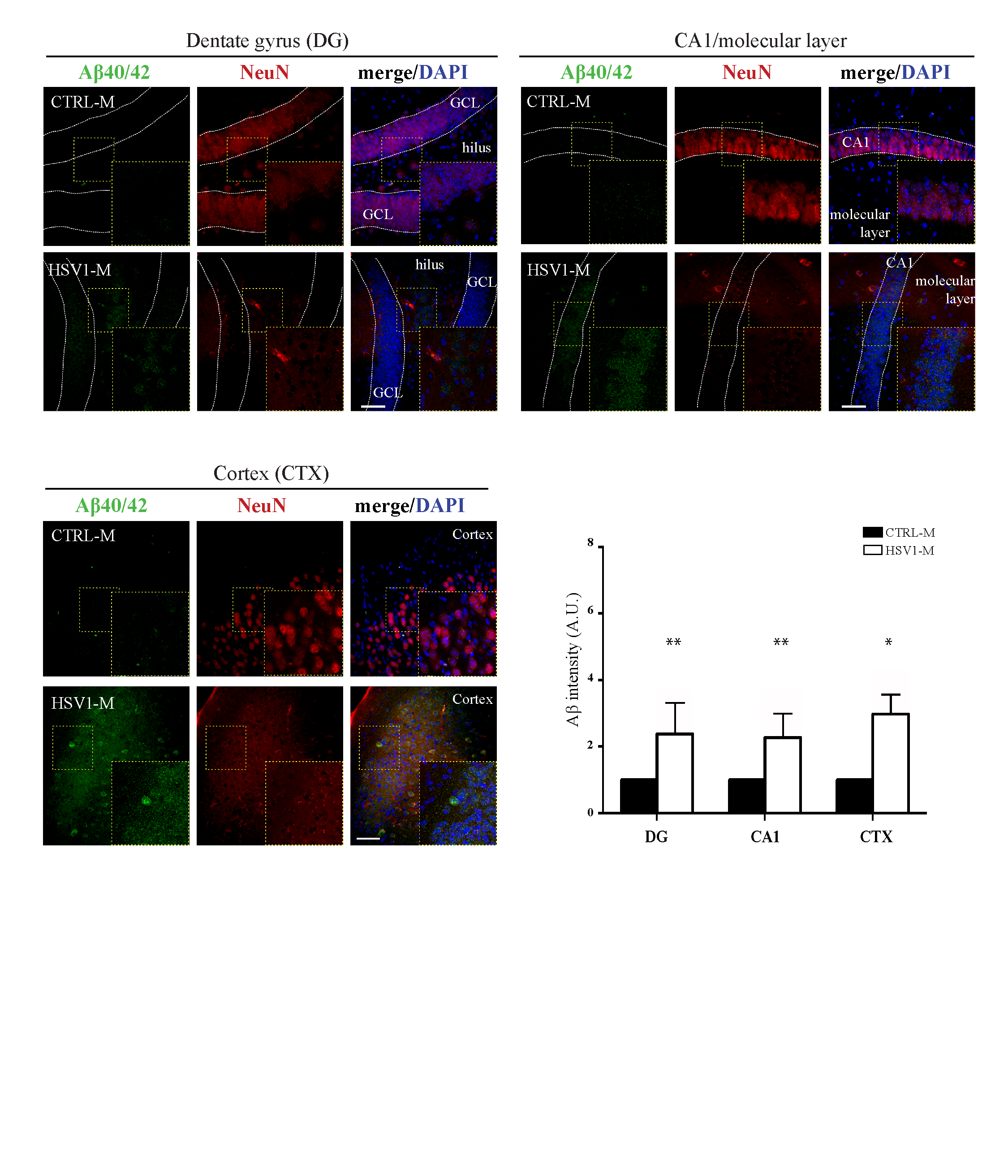

Supplement: S3 Fig — Confocal immunofluorescence analysis of coronal brain slices from HSV1-M and CTRL-M undergone 3 TSs (n = 2 mice for each experimental group). Two/three coronal sections were analyzed for each brain. Aβ40/42 were recognized by immunoreactivity for a specific antibody (see S7B Fig). Neurons were identified by their immunoreactivity for anti-NeuN antibody. Cell nuclei were stained with DAPI. Panels show representative images from the DG, CA1 and somatosensory neocortex (CTX). Insets show higher magnification (3×) of boxes outlined in each panel. Dotted lines delimitate pyramidal neuron layer in CA1 and granule cell layer (GCL) from hilus in DG. Scale bar: 50 μm. Bar graphs showing mean Aβ fluorescence intensity quantified in the studied brain areas and expressed as fold change with respect to CTRL-M. Data are represented as mean ± SEM, * p<0.05, ** p<0.01. (TIF) [file ppat.1007617.s003.tif]

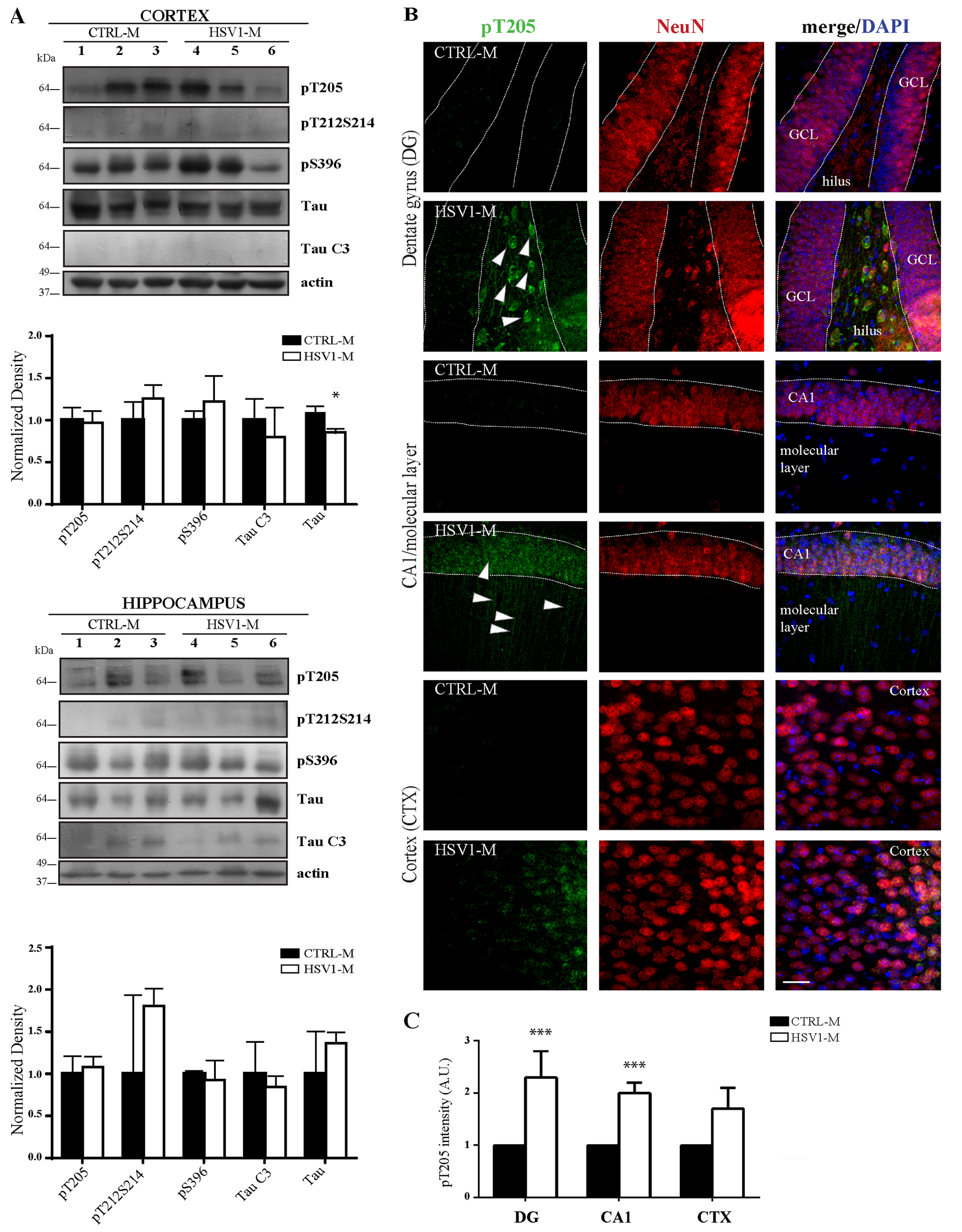

Supplement: S4 Fig — (A) Levels of phospho-tau and of its cleaved fragment TauC3 were investigated by using specific antibodies (see S7B Fig) in neocortex (upper gels) and hippocampus (bottom gels) homogenates from CTRL-M and HSV1-M sacrificed following 3 TSs (n = 3 for each experimental group). Actin expression level was used as sample loading control. Densitometric analysis of immunoreactive signals normalized to tau (for phospho-tau) or actin (for tau [Tau] and TauC3) are shown in the bar graphs: values represent the normalized fold changes in protein levels from HSV1-M with respect to CTRL-M (mean ± SEM, n = 3); * p<0.05 HSV1-M vs CTRL-M. (B) Confocal immunofluorescence analysis of coronal brain slices from HSV1-M and CTRL-M undergone 3 TSs (n = 2 mice for each experimental group). Two/three coronal sections were analyzed for each brain. Phosphorylation of tau at Thr205 (pT205) was studied by a specific antibody (see S7B Fig). Neurons were identified by their immunoreactivity for anti-NeuN antibody. Cell nuclei were stained with DAPI. Panels show representative images from dentate gyrus (DG), CA1 and somatosensory neocortex (CTX). Dotted lines delimitate pyramidal neuron layer in CA1 and granule cell layer (GCL) from hilus in DG. Arrowheads indicate pT205 in neurons of the hilus and in the axons of molecular layer. Scale bar: 50 μm. (C) Bar graphs showing pT205 fluorescence as the mean value of fluorescence quantified in DG and CA1 areas of the hippocampus and neocortex, expressed as fold changes with respect to CTRL-M. Values are expressed as mean ± SEM, *** p<0.001 (p = 0.000583 for DG and CA1, p = 0.20 for CTX, assessed by ANOVA on ranks). (TIF) [file ppat.1007617.s004.tif]

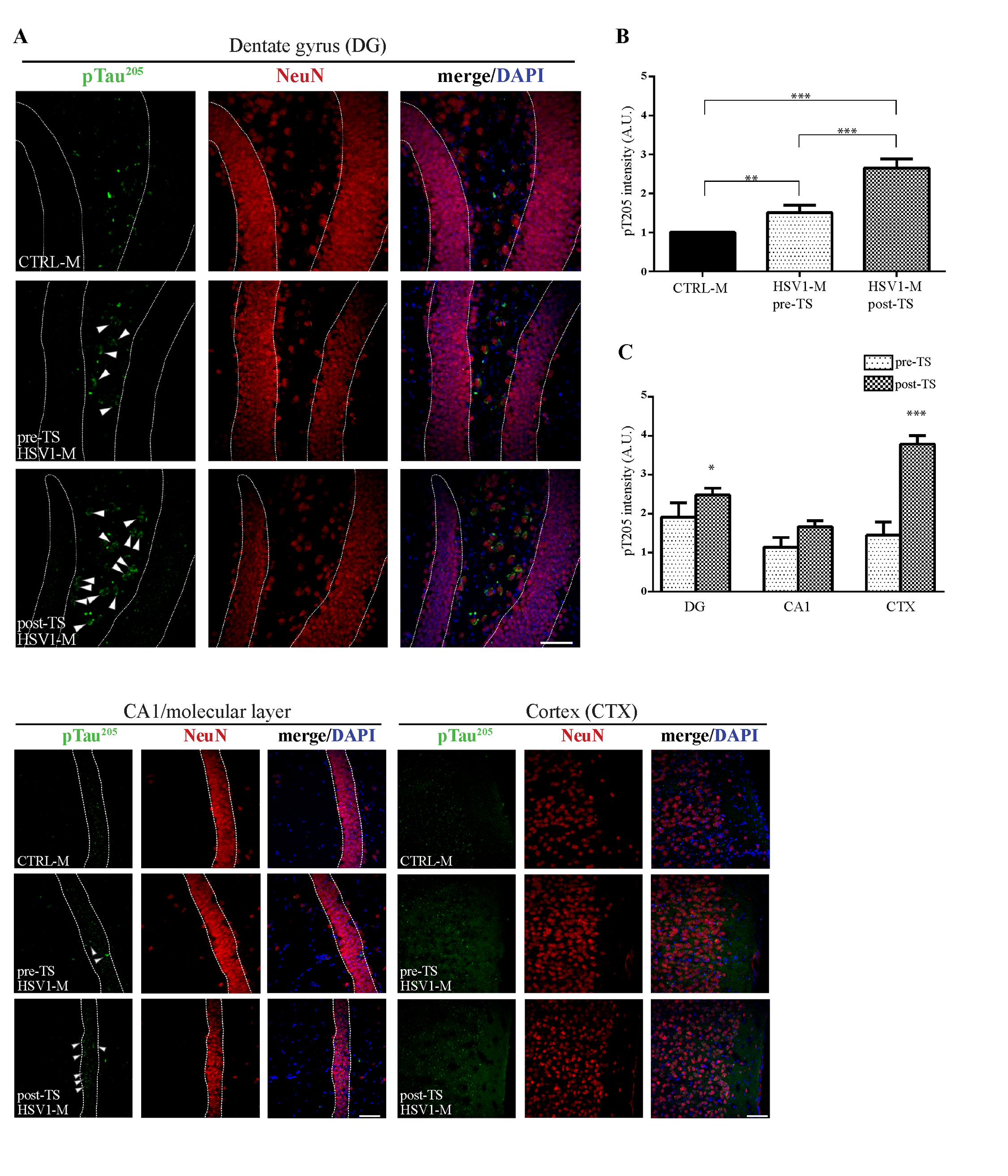

Supplement: S5 Fig — (A) Confocal immunofluorescence analysis on coronal brain slices from 4 HSV1-M and 2 CTRL-M undergone 6 TSs and sacrificed before the 7th TS (pre-TS) and from 3 HSV1-M and 1 CTRL-M sacrificed following the 7th TS (post-TS). Phosphorylation of tau at Thr205 (pT205) was studied by a specific antibody (see S7B Fig). Neurons were identified by their immunoreactivity for anti-NeuN antibody. Cell nuclei were stained with DAPI. Panels show representative images from dentate gyrus (DG), CA1/molecular layer and somatosensory neocortex (CTX). Dotted lines delimitate pyramidal neuron layer in CA1 and granule cell layer (GCL) from hilus in DG. Arrowheads indicate pT205 in neurons of the hilus and in CA1. (B) Bar graphs show pT205 intensity as the mean value of fluorescence quantified in the analyzed slices and expressed as fold changes with respect to CTRL-M. Values are expressed as mean ± SEM, *** p<0.001, ** p<0.01 (p<0.001 for post-TS vs pre-TS and vs CTRL-M; p = 0.001 for pre-TS vs CTRL-M, assessed by two-way ANOVA with Tukey post-hoc correction). (C) Bar graphs show pT205 intensity as the mean value of fluorescence quantified in the indicated brain areas and expressed as fold changes with respect to CTRL-M. Values are expressed as mean ± SEM, *** p<0.001, * p<0.05 vs pre-TS (p = 0.041 for DG and p<0.001 for CTX) (TIF) [file ppat.1007617.s005.tif]

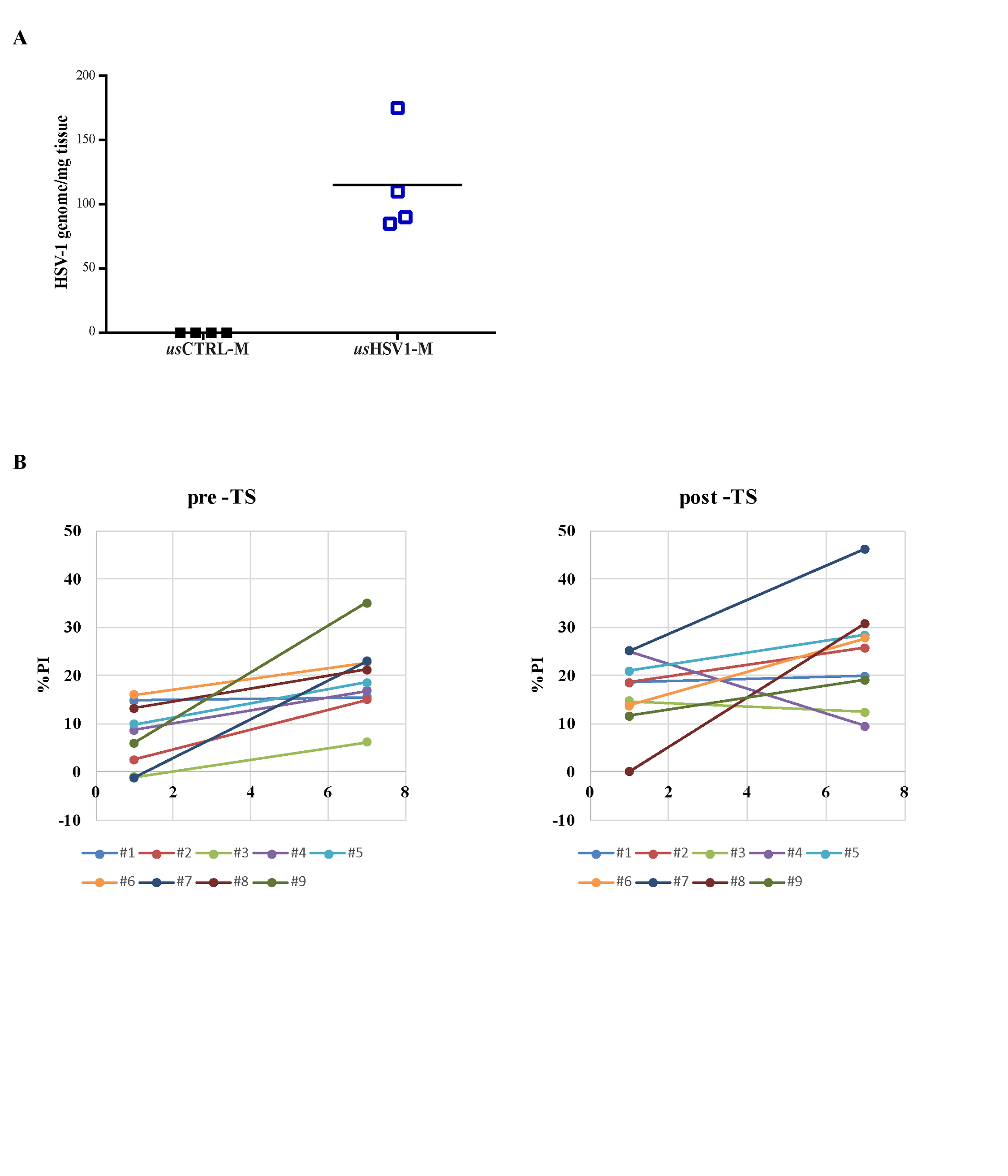

Supplement: S6 Fig — (A) Scatter dot plot showing qPCR quantification of the HSV-1 copies/mg in cortex homogenates from the 4 usHSV1-M analyzed in WB (see Fig 6B). Matched usCTRL-M were assessed as control. (B) Scatterplots showing individual %PI values of 9 HSV1-M vs matched CTRL-M tested in NOR one week before (left graph, pre-TS values) and one week after (right graph, post-TS values) the 1st and 7th TS. (TIF) [file ppat.1007617.s006.tif]

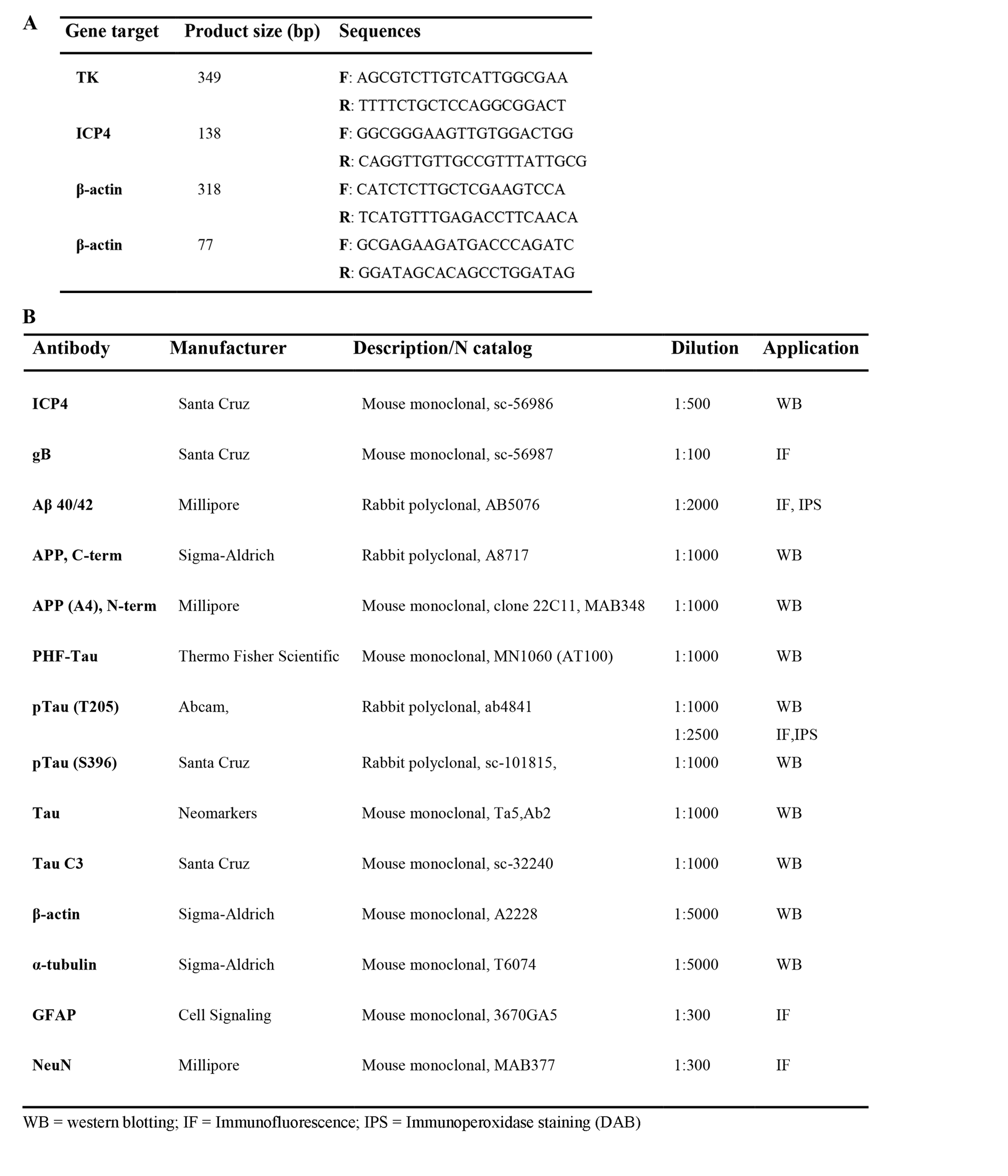

Supplement: S7 Fig — (A) Table summarizing the specific primers used for PCR detection of viral TK and cellular actin DNA, as well as for ICP4 and actin mRNA. (B) Table summarizing antibodies and their dilutions used for immunoblotting (WB), immunofluorescence (IF) or immunoperoxidase staining (IPS). (TIF) [file ppat.1007617.s007.tif]
